# The causal relationship between thyroid function, autoimune thyroid dysfunction and lung cancer: a mendelian randomization study

**DOI:** 10.1186/s12890-023-02588-0

**Published:** 2023-09-11

**Authors:** Xinhui Wang, Xue Liu, Yuchen Li, Mulin Tang, Xue Meng, Yuwei Chai, Li Zhang, Haiqing Zhang

**Affiliations:** 1 Department of Endocrinology, Shandong Provincial Hospital, Shandong University, Jinan, Shandong 250021 China; 2 Department of Endocrinology, Shandong Provincial Hospital Affiliated to Shandong First Medical University, Jinan, Shandong 250021 China; 3 Department of Vascular Surgery, Shandong Provincial Hospital Affiliated to Shandong First Medical University, Jinan, Shandong 250021 China; 4 Shandong Clinical Medical Center of Endocrinology and Metabolism, Jinan, 250021 China; 5 Institute of Endocrinology and Metabolism, Shandong Academy of Clinical Medicine, Jinan, 250021 China

**Keywords:** Thyroid function, Hypothyroidism, Hyperthyroidism, Lung cancer, Mendelian randomization

## Abstract

**Background:**

The role of thyroid hormones in cancers has been discussed in observational studies; however, the causal relationship between them remains controversial.

**Methods:**

The SNPs associated with hypothyroidism and hyperthyroidism were selected from a FinnGen biobank of 342,499 (190,879 females and 151,620 males) Finnish adult subjects. Data from the Thyroidomics Consortium on 72,167 individuals were used to assess genetically determined thyroid-stimulating hormone (TSH) and free thyroxine (FT4). Lung cancer, lung adenocarcinoma and squamous cell lung cancer GWAS data from the International Lung Cancer Consortium(ILCCO). Six different Mendelian randomization (MR) Methods, including Inverse variance weighted (IVW), MR-Egger, Simple mode, MR-Pleiotropy Residual Sum and Outlier methods (MR-PRESSO), Weighted mode and Weighted median were used to Two-Sample MR analysis. IVW was used as the primary estimate. Sensitivity analyses were examined via four aspects (Cochran’s Q-test, MR Egger intercept analysis, Funnel plot and Leave-one-out sensitivity test).

**Results:**

The OR of hypothyroidism on lung cancer was 0.918 (95% CI, 0.859–0.982; *p* = 0.013) in MR analysis with IVW method. No evidence for effects of hyperthyroidism, TSH and FT4 on lung cancer risk was found via six MR methods. Meanwhile, there was no evidence for effects of lung cancer on hypothyroidism through six MR methods. Lung adenocarcinoma and squamous cell lung carcinoma were further analyzed on the basis of lung cancer. The OR of hypothyroidism on lung adenocarcinoma was 0.893(95% CI, 0.813–0.981; *p* = 0.019), the OR of hypothyroidism on squamous cell lung cancer was 0.888(95%CI,0.797–0.990, *p* = 0.032) in MR analysis with IVW method.

**Conclusion:**

In summary, hypothyroidism genetically had a protective causal association with lung cancer. Furthermore, hypothyroidism had protective effects both on lung adenocarcinoma and squamous cell lung cancer. Further work is needed to elucidate the potential mechanisms.

**Supplementary Information:**

The online version contains supplementary material available at 10.1186/s12890-023-02588-0.

## Introduction

Lung cancer represents the most common malignancy and the leading cause of cancer death [1, 2], with age-standardized mortality of 18.0 per 100,000 people [2, 3]. Due to low adherence to lung cancer screening guidelines and because early-stage disease is typically asymptomatic [4], only 16% of lung cancers are detected at an early stages [5]. Therefore, it is significant to identify potential factors for better prevention. The relationship between thyroid hormones and cancers has been a hot topic for decades. The main cause of hypothyroidism in iodine-sufficient regions of the world is autoimmune hypothyroidism, also known as Hashimoto’s thyroiditis. The most common cause of hyperthyroidism is Graves’ disease (autoimmune hyperthyroidism) [6–8], which is caused by autoantibodies to the thyroid-stimulating hormone receptor (TSHR) that acts as agonists and induce excessive thyroid hormone secretion, releasing the thyroid gland from pituitary control [9]. Thyroid dysfunction may be involved in cancers [10].

A large number of prospective case-control studies reviewed that the increased risk of solid tumors may be associated with subclinical hyperthyroidism and Hashimoto’s thyroiditis may delay the incidence of cancers or reduce invasion of cancers [11]. Between 1995 and 1997, the site-specific analyses, starting follow-up 2 years after baseline, showed that subclinically hyperthyroidism people were at higher risk of lung cancer and prostate cancer [12]. From 1997 to 2000, one study had found that autoimmune hypothyroidism may be associated with reduced risk and severity of breast cancer [13], and it had been suggested that the presence of thyroid autoantibodies in patients with breast cancer, indicating autoimmune thyroiditis, may be associated with improved prognosis [14].

A nationwide cohort study found women with hyperthyroidism have a higher risk of breast cancer, while those with hypothyroidism had a slightly reduced risk of breast cancer which suggested thyroid hormone levels associated with breast cancer risk [15]. There are different opinions on the relationship between thyroid hormone levels and lung cancer. A prospective population-based cohort study showed that comparing the highest FT4 tertile to the lowest, the risk of lung cancer nearly doubled [16]. However, Ma Zhenchao et al.’s study in China showed that free tri-iodothyronine (FT3) in lung cancer patients was lower than that in healthy controls, and free thyroxine (FT4) was higher than that in healthy controls, with data from 2016 to 2021 [17]. Nevertheless, there was no association between thyroid hormone levels and cancer risk in other studies. A follow-up health survey was conducted in 1994/1995 among survivors who had participated in the 1966 Busselton Health Study survey. In this population of community-dwelling men and women, thyroid hormones including thyroid-stimulating hormone (TSH) and FT4 were not associated with incidence of lung cancer [18]. Due to adjused for different confounders, unmeasured confounders and measurements bias and/or inadequate power in the studies, there are different opinions on the relationship between thyroid hormone levels and cancers.

Lots of genome-wide association studies (GWASs) available on line has promoted a lower TSH and higher free thyroxine predict incidence of prostate but not breast, colorectal or lung cancer effective means to indicate the level or the biological effects of a modifiable environmental exposure which alters disease risk [19]. A widely-used method, Mendelian randomization (MR), can evaluate the causal relationship of exposure-outcome pair through instrumental single nucleotide polymorphisms (SNP) variables [20]. The association between hypothyroidism and breast cancer and hepatocellular carcinoma has been verified by MR analysis [21, 22]. However, the causal relationship between thyroid function and lung cancer remains unclear. In the present study, we used MR analysis, to investigate the causal relationship between thyroid function and lung cancer. Further analysis regarding the relationship between thyroid function and lung adenocarcinoma and squamous cell lung cancer was conducted to interpret the causal relationship between thyroid function and lung cancer.

## Materials and methods

### Data sources

Hypothyroidism and hyperthyroidism GWAS summary statistics data released FinnGen R8 alliance (https://www.finngen.fi/en). The phenotype used in this study was “hypothyroidism, strict autoimmune” and “autoimmune hyperthyroidism”. On the one hand, the hypothyroidism GWAS included 287,247 Finnish adult subjects, including 36,321 cases and 250,926 controls. Patients in the autoimmune hypothyroidism database include those who have already received treatment. On the other hand, the hyperthyroidism GWAS included 257,552 Finnish adult subjects, including 1,621 cases and 255,931 controls. The hyperthyroidism database does not specify whether the included patients have received treatments. Data from the Thyroidomics Consortium on 72,167 individuals were used to assess genetically determined TSH and FT4 [23]. The data included multiple cohorts, and TSH and FT4 in the cohorts were derived from their respective normal ranges. Lung cancer, lung adenocarcinoma and squamous cell lung cancer GWAS data from the International Lung Cancer Consortium study (http://ilcco.iarc.fr/) and the detailed information of the data is presented in the Related Manuscript. This GWAS included 27,209 European adult subjects, including 11,348 cases and 15,861 controls, 3,442 cases and 14,894 controls and 3,275 cases and 15,038 controls, respectively. All data sets used in this study were from public databases (listed in Supplementary Table S1).

### Selection of genetic instruments

**Fig. 1:**
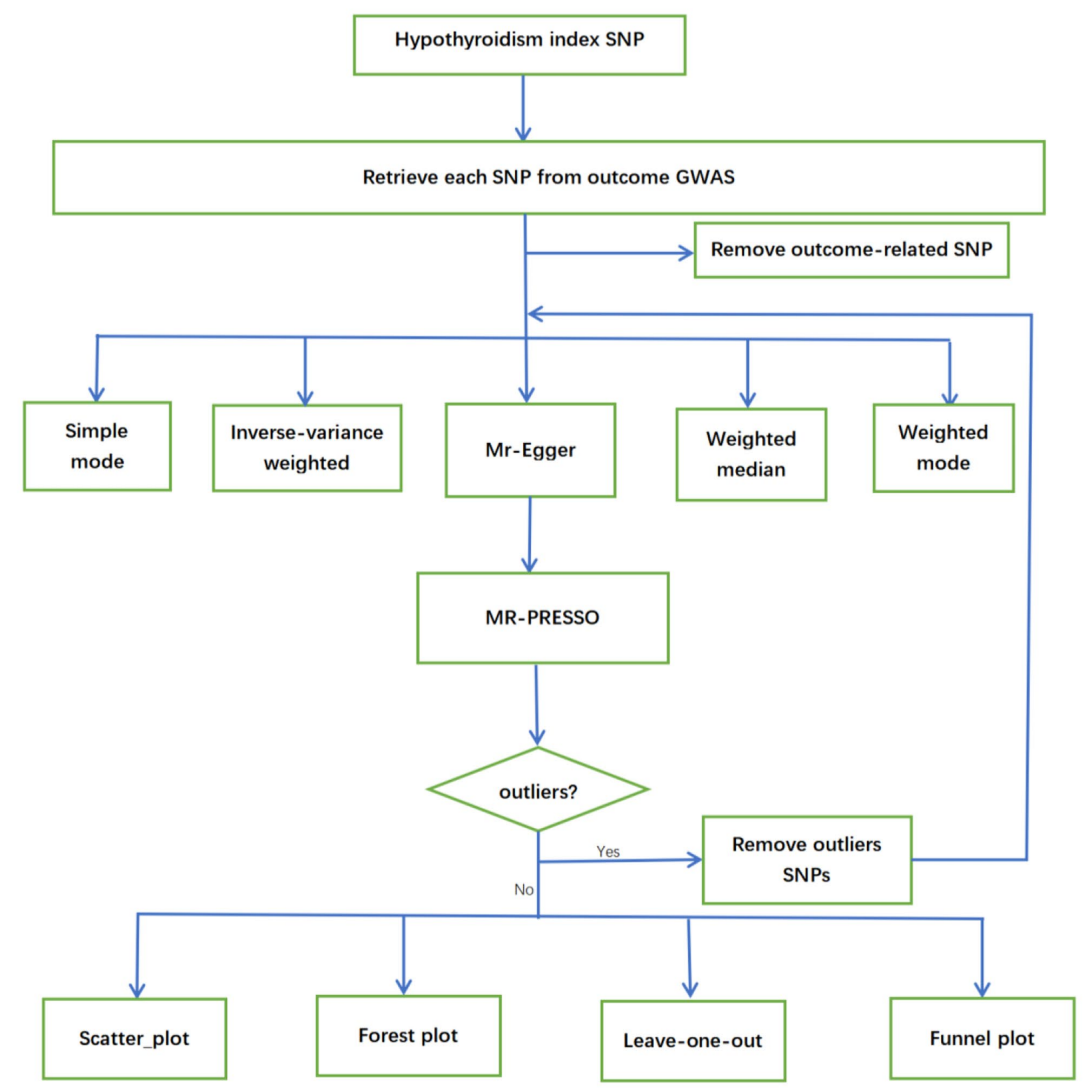
Study chart of the Mendelian randomization analysis revealing the causal relationship between hypothyroidism and lung cancer

Genetic instruments were selected via the following criteria: Genetic variation was associated with exposure; This genetic variation was not associated with any confounders of the expose-outcome association; The genetic variation didn’t affect the results unless possible through an association with exposure [24]. We followed the previous MR study with a stringent selection procedure [25]. Taking the causal effect of hypothyroidism on lung cancer from MR Analysis as an example, the others followed the same methods. We selected independent SNPs associated with hypothyroidism at genome-wide significance (*p* < 5 × 10^− 8^). Independence of SNPs was assessed using stringent criteria (r^2^ ≤ 0.001; clumping window, 10 000 kb). 100 independent instrumental SNPs were selected related with hypothyroidism. Then, independent instrumental SNPs were harmonised with the outcome lung cancer GWAS summary statistics. Removing the following SNPs for being palindromic with intermediate allele frequencies: rs116938742, rs12540388, rs12897126, rs140367581, rs151234, rs17061602, rs2757041, rs4743131, rs56308324, rs568999, rs7090504, rs74108950, rs7861040. After that only lefted 87 SNPs. We also checked in PhenoScanner (www.phenoscanner.medschl.cam.ac.uk [26, 27]), a platform with comprehensive information on the association of genotype and phenotype, to see whether these SNPs were associated with the potential risk factors, including smoking, radon gas, asbestos, vehicle exhaust, automobile exhaust and fuel burning. Through this website we found two SNPs associated with smoking, including rs3184504 and rs11086103. Then only lefted 85 SNPs (listed in Supplementary Table S2). These SNPs explained 1.939% of the variability in Hypothyroidism. The F statistic (also known as the Cragg-Donald statistic) was employed to evaluate the issue of weak instrument in MR analysis [25]. The F-statistics was 66.801, larger than the conventional value of 10 which indicated that the instruments had strong potential to predict hypothyroidism [28]. The assumptions and design of the MR study were shown in Fig. 1.

### Statistics

All analyses were performed using the packages TwoSampleMR (version 0.5.6) and MRPRESSO (version 1.0) in R (version 4.2.1) packages. For a global-level test, a significant two-sided P-value was set as 0.05. Six different MR Methods which included Inverse variance weighted (IVW), MR-Egger [29], MR-Pleiotropy Residual Sum and Outlier methods (MR-PRESSO), Simple mode [30], Weighted mode [30] and Weighted median [31] were used to Two-Sample MR analysis [32]. IVW was used as the main estimate [33], whereas MR-Egger, Simple mode, Weighted mode and Weighted median were lower than IVW (CIs) [29]. MR-Egger allows all genetic variants to have a pleiotropic effect, but requires that the pleiotropic effect is independent of the mutation-exposure association [29]. The Weighted median allows the use of invalid tools assuming that at least half of the tools used in the MR Analysis are valid. The causal effect was reported as odds ratios (ORs) that can be interpreted as the increase in lung cancer risk per unit increase in log odds of primary hypothyroidism. Sensitivity analyses were examined via four aspects (Cochran’s Q-test, MR Egger intercept analysis, Funnel plot and Leave-one-out sensitivity test). Cochran’s Q-test [34], Funnel plot and Leave-one-out sensitivity test were used to detect heterogeneity. Leave-one-out plot can see how each SNP affects the results and also assess the robustness of the results. If there is strong heterogeneity (*p* < 0.05) among these instrumental variables, at this time, we need to find outliers through MR-PRESSO [35] and eliminate them [36]. MR Egger intercept analysis was used to detect pleiotropy. There was no statistical difference between MR Egger intercept (*p* > 0.05), so we can assume that there is no horizontal pleiotropy. In the MR Egger intercept analysis, the slope of the weighted regression of the SNP outcome effect to the SNP exposure effect (where the intercept constraint is zero) represents the resulting estimate. Scatter plot, Forest plot, Funnel plot, and leave-one-out sensitivity test can realize the visualization of Mendelian randomization results.

## Results

### Causal effect of hypothyroidism on lung cancer from MR Analysis

Totally, 85 SNPs were selected as valid instrumental variables for hypothyroidism (listed in Supplementary Table S2). There were statistical indications of heterogeneity among instrumental variables (P-het = 0.024) [37]. However, the MR-PRESSO outlier test identified no outlier instrumental SNPs at the nominal significance level of 0.05. Both the funnel plot and leave-one-out plot also indicated the non-existence of outlier instrumental SNPs, strengthening the robustness of our results. Forest plot, leave-one-out plot, funnel plot and scatter plot were listed in Supplementary Figure S1.

**Fig. 2:**
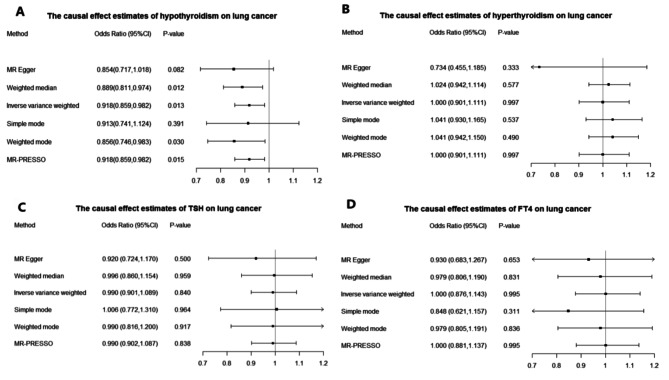
Odds ratios and confidence intervals (CI) correspond to the effects of hypothyroidism, hyperthyroidism, TSH and FT4 on lung cancer

In MR analysis with Inverse variance weighted (IVW) method, we found that hypothyroidism was negatively associated with lung cancer. The causal effect was reported as odds ratios (ORs) that can be interpreted as the increase in lung cancer risk per unit increase in log odds of primary hypothyroidism. The OR of hypothyroidism on lung cancer was estimated to be 0.918 (95% CI, 0.859–0.982; *p* = 0.013) in MR analysis with Inverse variance weighted (IVW) method, indicating that people with hypothyroidism can lead to an average of 8.2% decrease in the risk of lung cancer and suggesting hypothyroidism may play a protective role in the development of lung cancer. It was estimated to be 0.889 (95% CI, 0.811–0.974; *p* = 0.012) by the Weighted median method, 0.854 (95% CI, 0.717–1.018; *p* = 0.082) by MR-Egger method, 0.918 (95% CI, 0.859–0.982; *p* = 0.015) by MR-PRESSO method, 0.913 (95% CI, 0.741–1.124; *p* = 0.391) by Simple mode method and 0.856 (95% CI, 0.746–0.983; *p* = 0.030) by Weighted mode method (Fig. 2A). This was also confirmed by the OR values of all methods being less than 1.

### Causal effect of hyperthyroidism on lung cancer from MR Analysis

5 SNPs were selected as instrumental variables. There were statistical indications of heterogeneity among instrumental variables (P-het < 0.05). MR-PRESSO outlier test identified one outlier (rs75760731) instrumental SNPs at the nominal significance level of 0.05. Then lefted 4 SNPs, which together explained about 0.095% phenotypic variance of hyperthyroidism. There were no stastical indications of heterogeneity among instrumental varialbles (P-het > 0.05). The F statistics of hyperthyroidism was 61.209 (listed in Supplementary Table S3). Because the number of SNPs is too small, the funnel plot and leave-one-out plot cannot determine whether there are outliers. Forest plot, leave-one-out plot, funnel plot and scatter plot were listed in Supplementary Figure S2.

The Causal Effect of hyperthyroidism on lung cancer from MR Analysis was estimated to be 1.000 (95% CI, 0.901–1.111; *p* = 0.997) by the Inverse variance weighted (IVW) method, 1.024 (95% CI, 0.942–1.114; *p* = 0.577) by the Weighted median method, 0.734 (95% CI, 0.455–1.185; *p* = 0.333) by MR-Egger method, 1.000(95% CI, 0.901–1.111; *p* = 0.997) by MR-PRESSO method, 1.041 (95% CI, 0.930–1.165; *p* = 0.537) by Simple mode method and 1.041 (95% CI, 0.942–1.150; *p* = 0.490) by Weighted mode method (Fig. 2B).

### Causal effect of TSH and FT4 on lung cancer from MR Analysis

42 SNPs and 20 SNPs were selected as valid instrumental variables for TSH and FT4, which together explained about 6.930% phenotypic variance of TSH and 3.272% phenotypic variance of FT4. The F statistics of TSH was 96.166. The F statistics of FT4 was 83.302(listed in Supplementary Table S4 and S5). There were no statistical heterogeneities among the instrumental variables (P-het > 0.05) both TSH and FT4 on lung cancer From MR Analysis. The MR-PRESSO outlier test identified no outlier instrumental SNPs at the nominal significance level of 0.05. Both the funnel plot and leave-one-out plot also indicated the non-existence of outlier instrumental SNPs.

The Causal Effect of TSH on lung cancer from MR Analysis was estimated to be 0.990 (95% CI, 0.901–1.089; *p* = 0.840) by the Inverse variance weighted (IVW) method, 0.996 (95% CI, 0.860–1.154; *p* = 0.959) by the Weighted median method, 0.920 (95% CI, 0.724–1.170; *p* = 0.500) by MR-Egger method, 0.990 (95% CI, 0.902–1.087; *p* = 0.838) by MR-PRESSO method,1.006 (95% CI, 0.772–1.310; *p* = 0.964) by Simple mode method and 0.990 (95% CI, 0.816-1.200; *p* = 0.917) by Weighted mode method. (Fig. 2C). Forest plot, leave-one-out plot, funnel plot and scatter plot were listed in Supplementary Figure S3.

The Causal Effect of FT4 on lung cancer from MR Analysis was estimated to be 1.000 (95% CI, 0.876–1.143; *p* = 0.995) by the Inverse variance weighted (IVW) method, 0.979 (95% CI, 0.806–1.190; *p* = 0.831) by the Weighted median method, 0.930 (95% CI, 0.683–1.267; *p* = 0.653) by MR-Egger method, 1.000 (95% CI, 0.881–1.137; *p* = 0.995) by MR-PRESSO method, 0.848 (95% CI, 0.621–1.157; *p* = 0.311) by Simple mode method and 0.979 (95% CI, 0.805–1.191; *p* = 0.836) by Weighted mode method (Fig. 2D). Forest plot, leave-one-out plot, funnel plot and scatter plot were listed in Supplementary Figure S4.

### Causal effect of lung cancer on Hypothyroidism from MR Analysis

In addition, 5 SNPs were selected as valid instrumental variables for lung cancer. MR-PRESSO outlier test identified two outlier instrumental SNPs (rs8040868, rs501942) at the nominal significance level of 0.05. Then 3 SNPs were selected as valid instrumental variables for lung cancer (listed in Supplementary Table S6). There was no statistical heterogeneity among the instrumental variables (P-het > 0.05). There were not enough SNPs to do the MR-PRESSO, so we cannot determine whether there were outliers. Because the number of SNPs was too small, the funnel plot and leave-one-out plot cannot determine whether there were outliers. Forest plot, leave-one-out plot, funnel plot and scatter plot were listed in Supplementary Figure S5.

**Fig. 3:**
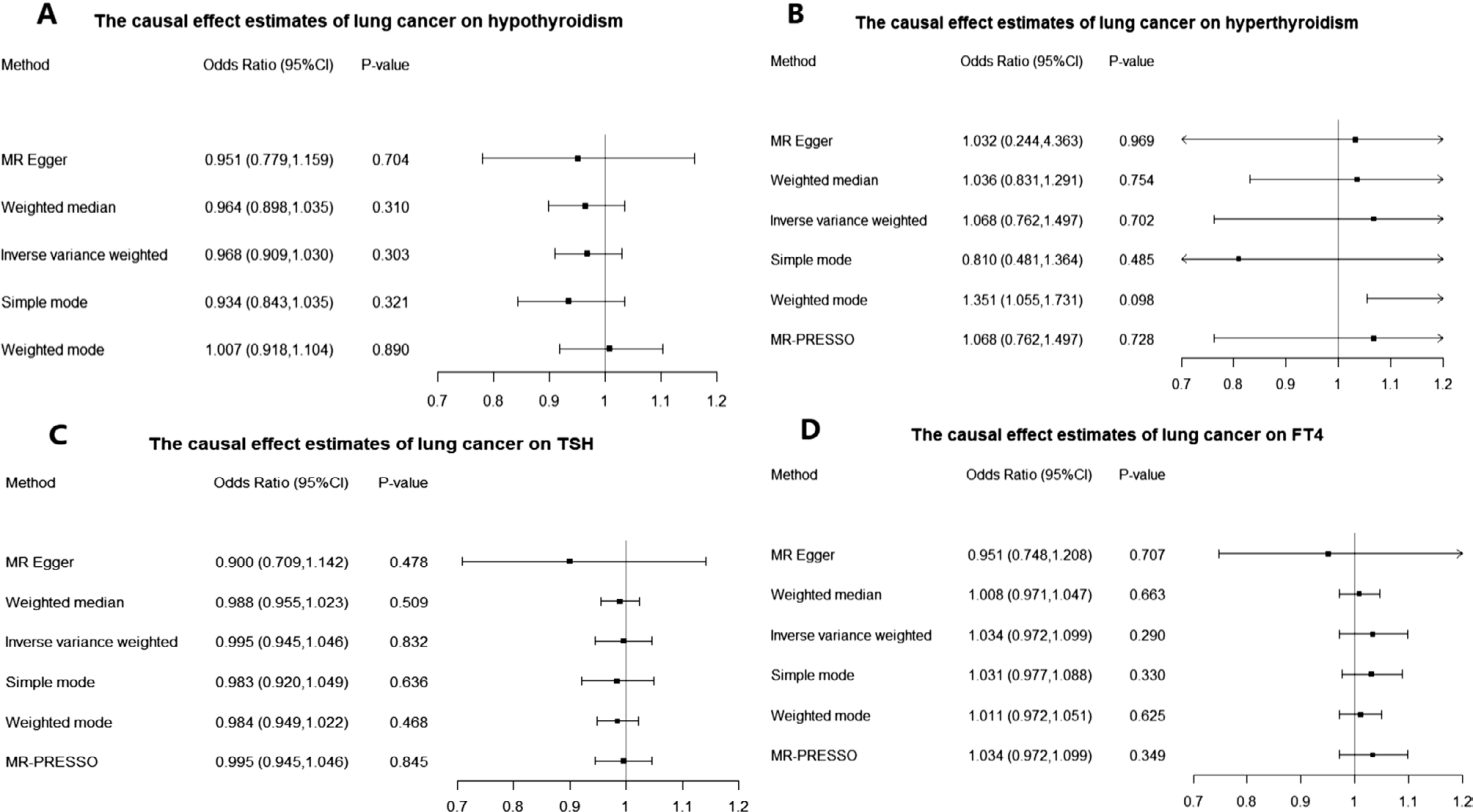
Odds ratios and confidence intervals (CI) correspond to the effects of lung cancer on hypothyroidism, hyperthyroidism, TSH and FT4.

In the MR analysis using lung cancer as exposure, there was no evidence for a direct causal effect of lung cancer level (OR = 0.968, 95% CI = 0.909–1.030, *p* = 0.303) on hypothyroidism risk by the Inverse variance weighted (IVW) method. It was estimated to be 0.964 (95% CI, 0.898–1.035; *p* = 0.310) by the Weighted median method, 0.951 (95% CI, 0.779–1.159; *p* = 0.704) by MR-Egger method, 0.934 (95% CI, 0.843–1.035; *p* = 0.321) by Simple mode method and 1.007 (95% CI, 0.918–1.104; *p* = 0.890) by Weighted mode method (Fig. 3A).

### Causal effect of lung cancer on hyperthyroidism from MR Analysis

4 SNPs were selected as instrumental variables (listed in Supplementary Table S7). There was statistical heterogeneity among the instrumental variables (P-het < 0.05). The MR-PRESSO outlier test identified no outlier instrumental SNPs at the nominal significance level of 0.05. Because the number of SNPS were too small, the funnel plot and leave-one-out plot cannot determine whether there were outliers. Forest plot, leave-one-out plot, funnel plot and scatter plot were listed in Supplementary Figure S6.

The Causal Effect of lung cancer on hyperthyroidism from MR Analysis was estimated to be 1.068 (95% CI, 0.762–1.497; *p* = 0.702) by the Inverse variance weighted (IVW) method, 1.036 (95% CI, 0.831–1.291; *p* = 0.754) by the Weighted median method, 1.032 (95% CI, 0.244–4.363; *p* = 0.969) by MR-Egger method, 1.068 (95% CI, 0.762–1.497; *p* = 0.728) by MR-PRESSO method, 0.810 (95% CI, 0.481–1.364; *p* = 0.485) by Simple mode method and 1.351 (95% CI, 1.055–1.731; *p* = 0.098) by Weighted mode method (Fig. 3B).

### Causal effect of lung cancer on TSH and FT4 from MR Analysis

The instrumental variables for causal effect of lung cancer on TSH and FT4 from MR analysis were 4 SNPs and 5 SNPs (listed in Supplementary Table S8 and S9). There were statistical heterogeneity among the instrumental variables (P-het < 0.05). The MR-PRESSO outlier test identified no outlier instrumental SNPs at the nominal significance level of 0.05. Because the number of SNPs were too small, the funnel plot and leave-one-out plot cannot determine whether there were outliers.

The Causal Effect of lung cancer on TSH from MR Analysis was estimated to be 0.995 (95% CI, 0.945–1.046; *p* = 0.832) by the Inverse variance weighted (IVW) method, 0.988 (95% CI, 0.955–1.023; *p* = 0.509) by the Weighted median method, 0.900 (95% CI, 0.709–1.142; *p* = 0.478) by MR-Egger method, 0.995 (95% CI, 0.945–1.046; *p* = 0.845) by MR-PRESSO method,0.983 (95% CI, 0.920–1.049; *p* = 0.636) by Simple mode method and 0.984 (95% CI, 0.949–1.022; *p* = 0.468) by Weighted mode method (Fig. 3C). Forest plot, leave-one-out plot, funnel plot and scatter plot were listed in Supplementary Figure S7.

The Causal Effect of lung cancer on FT4 from MR Analysis was estimated to be 1.034 (95% CI, 0.972–1.099; *p* = 0.290) by the Inverse variance weighted (IVW) method, 1.008 (95% CI, 0.971–1.047; *p* = 0.663) by the Weighted median method, 0.951 (95% CI, 0.748–1.208; *p* = 0.707) by MR-Egger method, 1.034 (95% CI, 0.972–1.099; *p* = 0.349) by MR-PRESSO method, 1.031 (95% CI, 0.977–1.088; *p* = 0.330) by Simple mode method and 1.011 (95% CI, 0.972–1.051; *p* = 0.625) by Weighted mode method (Fig. 3D). Forest plot, leave-one-out plot, funnel plot and scatter plot were listed in Supplementary Figure S8.

### Causal effect of hypothyroidism on lung adenocarcinoma from MR Analysis

85 SNPs were selected as instrumental variables (listed in Supplementary Table S10). There were no statistical heterogeneity among the instrumental variables (P-het > 0.05). The MR-PRESSO outlier test identified no outlier instrumental SNPs at the nominal significance level of 0.05. Funnel plot and leave-one-out plot also indicated the non-existence of outlier instrumental SNPs, strengthening the robustness of our results. Forest plot, leave-one-out plot, funnel plot and scatter plot were listed in Supplementary Figure S9.

**Fig. 4:**
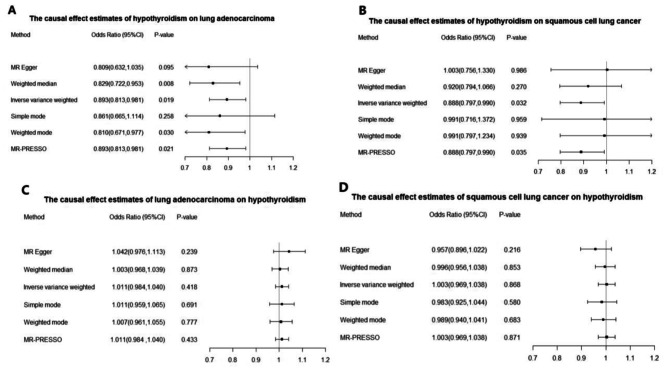
Odds ratios and confidence intervals (CI) correspond to the effects of hypothyroidism on lung adenocarcinoma and squamous cell lung cancer, lung adenocarcinoma and squamous cell lung cancer on hypothyroidism

The Causal Effect of hypothyroidism on lung adenocarcinoma from MR Analysis was estimated to be 0.893 (95% CI, 0.813–0.981; *p =* 0.019) by the Inverse variance weighted (IVW) method, suggesting hypothyroidism may play a protective role in the development of lung adenocarcinoma. It was estimated to be 0.829 (95% CI, 0.722–0.953; *p =* 0.008) by the Weighted median method, 0.809 (95% CI, 0.632–1.035; *p =* 0.095) by MR-Egger method, 0.893 (95% CI, 0.813–0.981; *p =* 0.021) by MR-PRESSO method, 0.861 (95% CI, 0.665–1.114; *p =* 0.258) by Simple mode method and 0.810 (95% CI, 0.671–0.977; *p =* 0.030) by Weighted mode method (Fig. 4A). This was also confirmed by the OR values of all methods being less than 1, strengthening the robustness of our results.

### Causal effect of hypothyroidism on squamous cell lung cancer from MR Analysis

85 SNPs were selected as instrumental variables (listed in Supplementary Table S11). There were statistical heterogeneity among the instrumental variables (P-het < 0.05). The MR-PRESSO outlier test identified no outlier instrumental SNPs at the nominal significance level of 0.05. Funnel plot and leave-one-out plot also indicated the non-existence of outlier instrumental SNPs, strengthening the robustness of our results. Forest plot, leave-one-out plot, funnel plot and scatter plot were listed in Supplementary Figure S10.

The Causal Effect of hypothyroidism on squamous cell lung cancer from MR Analysis was estimated to be 0.888 (95% CI, 0.797–0.990; *p =* 0.032) by the Inverse variance weighted (IVW) method. It was estimated to be 0.920 (95% CI, 0.794–1.066; *p =* 0.270) by the Weighted median method, 1.003 (95% CI, 0.756–1.330; *p =* 0.986) by MR-Egger method, 0.888 (95% CI, 0.797–0.990; *p =* 0.035) by MR-PRESSO method, 0.991 (95% CI, 0.716–1.372; *p =* 0.959) by Simple mode method and 0.991 (95% CI, 0.797–1.234; *p =* 0.939) by Weighted mode method (Fig. 4B). The results showed that hypothyroidism played a protective role in the development of squamous cell lung cancer.

### Causal effect of lung adenocarcinoma on Hypothyroidism from MR Analysis

In addition, 2 SNPs were selected as valid instrumental variables for lung adenocarcinoma, so the *p*-value was adjusted to 5 × 10^− 6^, 15 SNPs were selected. MR-PRESSO outlier test identified one outlier instrumental SNP (rs77468143) at the nominal significance level of 0.05. Then 14 SNPs were selected as instrumental variables (listed in Supplementary Table S12). There were no statistical heterogeneity among the instrumental variables (P-het > 0.05). The MR-PRESSO outlier test identified no outlier instrumental SNPs at the nominal significance level of 0.05. Funnel plot and leave-one-out plot also indicated the non-existence of outlier instrumental SNPs, strengthening the robustness of our results. Forest plot, leave-one-out plot, funnel plot and scatter plot were listed in Supplementary Figure S11.

In the MR analysis using lung adenocarcinoma as exposure, there was no evidence for a direct causal effect of lung adenocarcinoma level (OR = 1.011, 95% CI = 0.984–1.040, *p* = 0.418) on hypothyroidism risk by the Inverse variance weighted (IVW) method. It was estimated to be 1.003 (95% CI, 0.968–1.039; *p* = 0.873) by the Weighted median method, 1.042 (95% CI, 0.976–1.113; *p* = 0.239) by MR-Egger method, 1.011 (95% CI, 0.984–1.040; *p =* 0.433) by MR-PRESSO method, 1.011 (95% CI, 0.959–1.065; *p* = 0.691) by Simple mode method and 1.007 (95% CI, 0.961–1.055; *p* = 0.777) by Weighted mode method (Fig. 4C).

### Causal effect of squamous cell lung cancer on Hypothyroidism from MR Analysis

In addition, 3 SNPs were selected as valid instrumental variables for squamous cell lung cancer, so the p-value was adjusted to 5 × 10^− 6^, 13 SNPs were selected. ME-PRESSO outlier test identified one outlier instrumental SNP(rs501942) at the nominal significance level of 0.05. Then 12 SNPs were selected as instrumental variables (listed in Supplementary Table S13). There were no statistical heterogeneity among the instrumental variables (P-het > 0.05). The MR-PRESSO outlier test identified no outlier instrumental SNPs at the nominal significance level of 0.05. Funnel plot and leave-one-out plot also indicated the non-existence of outlier instrumental SNPs, strengthening the robustness of our results. Forest plot, leave-one-out plot, funnel plot and scatter plot were listed in Supplementary Figure S12.

In the MR analysis using squamous cell lung cancer as exposure, there was no evidence for a direct causal effect of squamous cell lung cancer level (OR = 1.003, 95% CI = 0.969–1.038, *p* = 0.868) on hypothyroidism risk by the Inverse variance weighted (IVW) method. It was estimated to be 0.996 (95% CI, 0.956–1.038; *p* = 0.853) by the Weighted median method, 0.957 (95% CI, 0.896–1.022; *p* = 0.216) by MR-Egger method, 1.003 (95% CI, 0.969–1.038; *p =* 0.871) by MR-PRESSO method, 0.983 (95% CI, 0.925–1.044; *p* = 0.580) by Simple mode method and 0.989 (95% CI, 0.940–1.041; *p* = 0.683) by Weighted mode method (Fig. 4D).

## Discussion

In the present study, we used the MR approaches to estimate the causal relationship between thyroid function and lung cancer. Our study demonstrated that hypothyroidism genetically had a protective causal association with lung cancer. Lung adenocarcinoma and squamous cell lung carcinoma were further analyzed on the basis of lung cancer. We found that hypothyroidism had protective effects on lung adenocarcinoma and squamous cell lung cancer.

Some studies suggested TH and TH receptors can promote cancers through mediating by phosphatidylinositol-3-kinase and MAPK [38]. TH receptors also contribute to proliferation of integrin αvβ3-bearing cells which usually include cancer cells and stimulate angiogenesis [38–40]. Thyroid hormones and TSH can promote cancer proliferation through TH receptors, increased angiogenesis, gene expression regulation and estrogen pathways [38, 41]. Gionfra et al. demonstrated that elevated rT3 might lead to cancer cell growth [42]. rT3 has received more and more attention in recent years [21]. Metabolites, when aberrantly accumulated, can also promote tumorigenesis [43]. Because thyroid hormone plays an important role in cell metabolism, thyroid hormone may affect the occurrence and development of lung cancer through cell metabolism.

In clinical studies, hyperthyroidism is correlated with cancer prevalence in various cancer types, including liver, thyroid, brain, breast, lung, and colorectal cancer [44, 45]. However, no such relationship between autoimmune hyperthyroidism and lung cancer was found in our Mendelian study. The reason may be that clinical research is interfered by many confounding factors. Whether hyperthyroidism can directly promote the occurrence of lung cancer needs further study.

Moreover, studies using continuous exposures (TSH and FT4) are considered necessary. TSH which is a sensitive marker could reflect both subclinical hypothyroidism and subclinical hyperthyroidism [46]. Data showed that TSH level (OR = 0.990, 95% CI = 0.901–1.089, *p* = 0.840) and FT4 level (OR = 1.000, 95% CI = 0.876–1.143, *p* = 0.995) have no significant causal relationship with lung cancer. There were several reasons. Firstly, our data on TSH and FT4 were obtained from the Thyroid Consortium. The data included multiple cohorts, and TSH and FT4 in the cohorts were derived from their respective normal ranges. However, TSH and FT4 in hypothyroidism may not be in this range. Secondly, the sample size of TSH and FT4 was relatively small, and the sample size may affect the results of the study. Further research is needed to expand our sample size for further research.

One strength of our study was that we used MR approaches to demonstrate hypothyroidism had a protective causal association with lung cancer. A variety of sensitivity analyses were employed to ensure consistency and robustness of our results. More importantly, we used multiple models with different model assumptions to guard against false positives due to model misspecifications. Besides, our data on autoimmune hyperthyroidism and autoimmune hypothyroidism were from the FinnGen R8 alliance which was FinnGen GWAS results from the latest public release. However, this study had several limitations. Firstly, we were unable to confirm whether TSH and FT4 in hypothyroidism and hyperthyroidism patients at the genetic level had any effects on lung cancer. Secondly, the data of our research from European ethnicity, the results cannot be generalized to other races. Finally, the GWAS sample size of lung adenocarcinoma and squamous cell lung cancer were relatively small.

## Conclusion

Our study is the first to demonstrate that hypothyroidism genetically has a protective causal relationship on lung cancer by Mendelian analysis methods. Furthermore, hypothyroidism has protective effects on lung adenocarcinoma and squamous cell lung cancer. Lung cancer, lung adenocarcinoma and squamous cell lung cancer have no effects on hypothyroidism. In addition, there are no causal effects between hyperthyroidism, TSH, FT4 in normal range and lung cancer. In addition, more researches are needed to demonstrate how this possible cause-and-effect relationship works, and prospective cohort studies are needed to determine whether hypothyroidism is helpful for lung cancer. Our study suggests whether patients with hypothyroidism at high risk of lung cancer are actively supplemented with thyroid hormone needs to be further discussed.

### Electronic supplementary material

Below is the link to the electronic supplementary material.

Supplementary Material 1

## Data Availability

All GWAS summary datasets analyzed during this study are publicly available. Hypothyroidism and hyperthyroidism GWAS summary statistics data released FinnGen R8 alliance (https://www.finngen.fi/en). GWAS summary data of TSH and FT4 can be available from the obtained from the Thyroid Omics Consortium study (https://transfer.sysepi.medizin.uni-greifswald.de/thyroidomics/datasets/). Lung cancer, lung adenocarcinoma and squamous cell lung cancer GWAS data from the International Lung Cancer Consortium study (http://ilcco.iarc.fr/).
