# Supplementary material for: The causal relationship between thyroid function, autoimune thyroid dysfunction and lung cancer: a mendelian randomization study
Source: BMC Pulm Med. 2023 Sep 11;23:338. doi: 10.1186/s12890-023-02588-0 (PMC10494366; doi:10.1186/s12890-023-02588-0)

**Keywords:** thyroid function; hypothyroidism; hyperthyroidism; lung cancer; Mendelian randomization

**Supplementary Material**

# The causal relationship between Thyroid function and Lung cancer: A mendelian randomization study

**Xinhui Wang^1^ ,Xue Liu^1^****,Yuchen Li^1^, Mulin Tang^2^, Xue Meng^2^, Yuwei Chai^1^, Li Zhang^3^ and Haiqing Zhang^1,2,4,5*^**

*** Correspondence:** Haiqing Zhang, Email: [zhanghq@sdu.edu.cn](mailto:zhanghq@sdu.edu.cn)；Li Zhang, Email:[lizhang7675@163.com](mailto:lizhang7675@163.com)

# 1 Supplementary Figures and Tables

## 1.1Supplementary Tables

**Supplementary Table S1** Detailed description of the GWAS database in this study

| Phenotype | Ancestry | N | Cases | Controls |
| --- | --- | --- | --- | --- |
| hypothyroidism | European | 287,247 | 36,321 | 250,926 |
| hyperthyroidism | European | 257,552 | 1,621 | 255,931 |
| TSH | European | 72,167 | - | - |
| FT4 | European | 72,167 | - | - |
| lung cancer | European | 27,209 | 11,348 | 15,861 |
| lung adenocarcinoma | European | 18,336 | 3,442 | 14,894 |
| squamous cell lung cancer | European | 18,313 | 3,275 | 15,038 |

Note：TSH and FT4 GWAS database origin from GWAS meta-analysis summary statistics，so N is a rough number. The data in bold in the table is the GWAS data used in the article

**Supplementary Table S2.** The instrumental SNP variables for hypothyroidism on lung cancer

| rsid | A1 | A2 | hypothyroidism | | | | | | | lung cancer | | |
| --- | --- | --- | --- | --- | --- | --- | --- | --- | --- | --- | --- | --- |
|  |  |  | P.x | beta.x | se.x | eaf | N | PVE | F | P.y | beta.y | se.y |
| rs10008892 | A | C | 1.06E-30 | -0.151395 | 0.0131437 | 0.123091 | 287247 | 0.00046167 | 132.6736768 | 0.588474976 | 0.0124887 | 0.022851 |
| rs10112995 | A | G | 1.32E-08 | 0.0483905 | 0.00851337 | 0.612318 | 287247 | 0.000112464 | 32.30830528 | 0.600222125 | 0.0100089 | 0.018558 |
| rs10118880 | A | G | 4.57E-13 | -0.0666212 | 0.00920486 | 0.723956 | 287247 | 0.000182329 | 52.38265457 | 0.014927944 | -0.0484834 | 0.020501 |
| rs10166287 | G | T | 2.21E-10 | -0.0881637 | 0.0138928 | 0.102756 | 287247 | 0.000140179 | 40.27142756 | 0.891462954 | 0.00367325 | 0.026323 |
| rs10259879 | G | A | 6.27E-09 | 0.0571615 | 0.00983933 | 0.232158 | 287247 | 0.000117482 | 33.74995177 | 0.637727287 | 0.0103978 | 0.021834 |
| rs10481413 | T | C | 1.28E-09 | -0.050671 | 0.008348 | 0.444792 | 287247 | 0.000128246 | 36.84266574 | 0.86018091 | 0.00325529 | 0.018092 |
| rs10494077 | A | G | 2.27E-28 | 0.110234 | 0.00997883 | 0.213359 | 287247 | 0.000424651 | 122.0306323 | 0.842186935 | -0.00435748 | 0.021331 |
| rs10748781 | A | C | 2.88E-20 | -0.0801497 | 0.00869 | 0.652669 | 287247 | 0.00029606 | 85.06702123 | 0.430080746 | 0.0152598 | 0.01869 |
| rs1079418 | G | A | 2.66E-19 | -0.0852228 | 0.00948814 | 0.261894 | 287247 | 0.000280784 | 80.67638048 | 0.326042339 | 0.0194271 | 0.019783 |
| rs10814915 | C | T | 3.58E-17 | -0.0698807 | 0.00829364 | 0.543644 | 287247 | 0.000247093 | 70.99391223 | 0.648083553 | 0.00833968 | 0.017798 |
| rs10858026 | G | T | 4.07E-10 | 0.0666008 | 0.010654 | 0.807372 | 287247 | 0.000136025 | 39.07783394 | 0.704558405 | -0.00836392 | 0.021769 |
| rs10948107 | A | G | 1.07E-10 | -0.0672386 | 0.0104134 | 0.202244 | 287247 | 0.000145122 | 41.69166111 | 0.157630045 | -0.0302005 | 0.020308 |
| rs11079035 | A | G | 4.69E-13 | 0.0738873 | 0.0102138 | 0.202278 | 287247 | 0.00018215 | 52.33134184 | 0.51704102 | 0.0156479 | 0.02396 |
| rs111352680 | A | G | 1.23E-08 | -0.0509487 | 0.00894548 | 0.323629 | 287247 | 0.000112916 | 32.43813614 | 0.362443237 | 0.0195094 | 0.021392 |
| rs11571297 | C | T | 4.06E-45 | -0.121325 | 0.0086075 | 0.373405 | 287247 | 0.000691179 | 198.6751334 | 0.183074982 | -0.0243921 | 0.017563 |
| rs116776245 | A | G | 3.54E-09 | 0.0869905 | 0.014733 | 0.0833056 | 287247 | 0.000121354 | 34.86247795 | 0.124472095 | -0.0636184 | 0.037324 |
| rs11683207 | C | T | 8.36E-09 | -0.0536415 | 0.0093111 | 0.278037 | 287247 | 0.00011553 | 33.18920286 | 0.358066755 | 0.0224804 | 0.024427 |
| rs11969311 | C | A | 1.22E-08 | -0.0545027 | 0.00956667 | 0.252339 | 287247 | 0.000112982 | 32.45722783 | 0.860122087 | -0.00399999 | 0.022115 |
| rs1203943 | C | T | 1.83E-23 | 0.103531 | 0.010372 | 0.79178 | 287247 | 0.000346745 | 99.63519559 | 0.183314154 | -0.0289441 | 0.021921 |
| rs12091047 | T | C | 1.08E-25 | 0.0927867 | 0.00885449 | 0.316692 | 287247 | 0.00038214 | 109.8098582 | 0.202949039 | -0.0241687 | 0.01819 |
| rs12129508 | A | G | 4.49E-08 | 0.0690302 | 0.0126184 | 0.120108 | 287247 | 0.000104176 | 29.92723948 | 0.85369296 | 0.00485918 | 0.02581 |
| rs12206423 | T | C | 1.24E-08 | 0.0655998 | 0.0115195 | 0.14809 | 287247 | 0.000112884 | 32.429089 | 0.005734955 | 0.0748579 | 0.028433 |
| rs123378 | A | G | 3.85E-09 | -0.0502193 | 0.00852516 | 0.617779 | 287247 | 0.000120789 | 34.70029834 | 0.584332443 | 0.010791 | 0.019141 |
| rs12506688 | T | C | 5.20E-14 | 0.0687936 | 0.00913992 | 0.281399 | 287247 | 0.000197183 | 56.65108878 | 0.114238108 | 0.0312913 | 0.02005 |
| rs12697352 | A | G | 1.49E-09 | -0.0514445 | 0.00850986 | 0.395739 | 287247 | 0.00012721 | 36.54517506 | 0.259762228 | 0.0226329 | 0.020144 |
| rs12736474 | A | G | 2.35E-09 | 0.053367 | 0.0089364 | 0.31216 | 287247 | 0.00012414 | 35.6629575 | 0.034800862 | -0.0413378 | 0.018434 |
| rs12756019 | A | G | 2.38E-11 | -0.0566832 | 0.00848487 | 0.60567 | 287247 | 0.000155344 | 44.6288104 | 0.370584286 | 0.0173425 | 0.01868 |
| rs13137589 | G | A | 4.42E-13 | -0.0627853 | 0.00866943 | 0.368031 | 287247 | 0.000182558 | 52.4483506 | 0.012207873 | 0.0472843 | 0.019416 |
| rs1317983 | C | T | 1.25E-27 | 0.0980504 | 0.00900134 | 0.680676 | 287247 | 0.000412904 | 118.6537267 | 0.820232928 | -0.00473378 | 0.020509 |
| rs13220783 | T | G | 3.99E-09 | 0.0499938 | 0.00849568 | 0.380325 | 287247 | 0.000120539 | 34.62844433 | 0.798020963 | 0.00478653 | 0.018455 |
| rs13447704 | C | T | 2.09E-08 | -0.14695 | 0.0262205 | 0.0268588 | 287247 | 0.000109334 | 31.40900791 | 0.979383989 | -0.00174051 | 0.063009 |
| rs1371867 | C | A | 5.71E-09 | 0.0487005 | 0.00836052 | 0.428582 | 287247 | 0.000118112 | 33.93104507 | 0.191964976 | 0.0239333 | 0.018454 |
| rs141686764 | G | A | 9.11E-09 | -0.182081 | 0.0316857 | 0.0187788 | 287247 | 0.000114947 | 33.02171493 | 0.485935917 | 0.0461963 | 0.065109 |
| rs1441172 | T | C | 3.43E-09 | -0.0533644 | 0.00903023 | 0.313428 | 287247 | 0.000121562 | 34.92228191 | 0.027377892 | 0.0409941 | 0.019009 |
| rs1534430 | T | C | 2.76E-11 | -0.056034 | 0.00841488 | 0.417775 | 287247 | 0.000154342 | 44.34088669 | 0.684863467 | -0.00754136 | 0.01811 |
| rs1788105 | G | A | 2.77E-08 | -0.0462256 | 0.00832056 | 0.547645 | 287247 | 0.000107438 | 30.86433873 | 0.059332138 | 0.0341557 | 0.017197 |
| rs2104047 | C | T | 1.20E-08 | 0.0514534 | 0.00902662 | 0.693491 | 287247 | 0.000113103 | 32.49187785 | 0.745672282 | -0.00639352 | 0.019459 |
| rs2111485 | G | A | 5.67E-18 | 0.0727979 | 0.00842671 | 0.583695 | 287247 | 0.000259749 | 74.63088343 | 0.03117023 | -0.0400338 | 0.018987 |
| rs2235544 | A | C | 4.66E-10 | -0.0519065 | 0.0083313 | 0.5448 | 287247 | 0.000135115 | 38.81637023 | 0.812521946 | -0.00430173 | 0.017896 |
| rs229528 | T | C | 6.96E-21 | 0.0790766 | 0.0084354 | 0.396388 | 287247 | 0.000305841 | 87.87828838 | 0.163576157 | -0.0253999 | 0.017461 |
| rs2402240 | A | G | 1.92E-18 | 0.0940805 | 0.0107375 | 0.176345 | 287247 | 0.000267191 | 76.76970224 | 0.578117346 | -0.0127904 | 0.0222 |
| rs244689 | G | A | 3.29E-14 | -0.0718721 | 0.00947387 | 0.749122 | 287247 | 0.00020032 | 57.55231573 | 0.432201231 | -0.0201418 | 0.025519 |
| rs2476601 | G | A | 1.71E-162 | -0.304028 | 0.0111921 | 0.854687 | 287247 | 0.002562325 | 737.9058911 | 0.041327582 | 0.0629654 | 0.028117 |
| rs2531989 | A | G | 5.90E-11 | -0.0695526 | 0.010625 | 0.817382 | 287247 | 0.000149159 | 42.85148111 | 0.880265944 | 0.00370385 | 0.023922 |
| rs2680296 | A | C | 1.17E-08 | 0.0473154 | 0.00829442 | 0.468365 | 287247 | 0.000113273 | 32.54093014 | 0.294018732 | -0.0193439 | 0.017759 |
| rs28391281 | C | T | 3.99E-09 | -0.0488629 | 0.00830364 | 0.546421 | 287247 | 0.000120535 | 34.627284 | 0.249210881 | -0.0237928 | 0.019762 |
| rs28582094 | G | A | 3.81E-09 | 0.0552873 | 0.00938328 | 0.261193 | 287247 | 0.000120846 | 34.71669602 | 0.426165217 | 0.016807 | 0.02104 |
| rs2928167 | G | A | 3.52E-22 | -0.101973 | 0.0105297 | 0.199071 | 287247 | 0.000326393 | 93.78542973 | 0.573993534 | 0.0152708 | 0.026858 |
| rs2958154 | T | C | 7.35E-12 | -0.0616386 | 0.0089973 | 0.699391 | 287247 | 0.000163363 | 46.93297725 | 0.788983644 | 0.00517336 | 0.018872 |
| rs30233 | A | G | 4.58E-12 | -0.0582169 | 0.00841513 | 0.586872 | 287247 | 0.00016659 | 47.86008537 | 0.927492059 | 0.00168842 | 0.018198 |
| rs35717611 | T | C | 1.26E-08 | 0.0487793 | 0.0085697 | 0.378643 | 287247 | 0.000112781 | 32.39939242 | 0.415108857 | 0.01761 | 0.021534 |
| rs3946137 | G | A | 1.22E-09 | 0.0526375 | 0.00866007 | 0.349188 | 287247 | 0.000128599 | 36.94406503 | 0.39643866 | 0.0171422 | 0.020162 |
| rs41177 | A | G | 2.48E-09 | -0.0504861 | 0.00846668 | 0.404374 | 287247 | 0.000123768 | 35.55611735 | 0.33419581 | 0.0180197 | 0.018656 |
| rs4409785 | C | T | 9.15E-16 | 0.0882346 | 0.0109776 | 0.165925 | 287247 | 0.000224859 | 64.60409017 | 0.346863019 | -0.0234027 | 0.02372 |
| rs4575545 | A | G | 1.12E-20 | -0.0852985 | 0.00914861 | 0.302816 | 287247 | 0.000302542 | 86.92997119 | 0.599796601 | 0.0104829 | 0.019798 |
| rs4606850 | T | C | 2.55E-08 | 0.0616895 | 0.0110754 | 0.165369 | 287247 | 0.000107994 | 31.0242032 | 0.615365296 | 0.0118851 | 0.02339 |
| rs4704397 | A | G | 8.89E-56 | 0.131555 | 0.00836138 | 0.41002 | 287247 | 0.000861052 | 247.5459192 | 0.510328708 | 0.0123554 | 0.018655 |
| rs4804433 | T | G | 2.49E-17 | -0.0754197 | 0.00890613 | 0.692424 | 287247 | 0.00024959 | 71.71145216 | 0.475833484 | -0.015704 | 0.021897 |
| rs4853459 | C | T | 8.01E-43 | -0.132855 | 0.0096853 | 0.769419 | 287247 | 0.000654621 | 188.1597073 | 0.062258693 | 0.0406387 | 0.020487 |
| rs56983610 | A | C | 5.30E-18 | -0.108696 | 0.0125708 | 0.13032 | 287247 | 0.000260215 | 74.76498787 | 0.533565819 | 0.0151309 | 0.024095 |
| rs57652885 | T | C | 2.65E-09 | -0.112845 | 0.0189593 | 0.0530945 | 287247 | 0.000123314 | 35.42558418 | 0.768884312 | 0.0216587 | 0.070136 |
| rs61759532 | T | C | 1.20E-12 | 0.0747524 | 0.0105202 | 0.188962 | 287247 | 0.00017574 | 50.48929016 | 0.503240981 | -0.0172621 | 0.024716 |
| rs61916675 | G | A | 3.01E-11 | 0.0590178 | 0.00888003 | 0.316646 | 287247 | 0.00015375 | 44.17068586 | 0.673604531 | 0.00823202 | 0.019332 |
| rs6724363 | T | G | 7.67E-24 | 0.0864868 | 0.00859042 | 0.624805 | 287247 | 0.000352746 | 101.3602885 | 0.666808305 | 0.00872193 | 0.019688 |
| rs6831973 | C | T | 4.47E-12 | 0.0577679 | 0.00834607 | 0.555424 | 287247 | 0.000166756 | 47.90778488 | 0.279078256 | 0.0197712 | 0.017593 |
| rs6914622 | T | G | 4.25E-14 | 0.0716868 | 0.00949102 | 0.254521 | 287247 | 0.000198569 | 57.04920385 | 0.624535314 | -0.00971201 | 0.019273 |
| rs7043516 | C | A | 1.45E-18 | -0.0998544 | 0.0113558 | 0.166059 | 287247 | 0.000269108 | 77.32072524 | 0.967631059 | -0.00107458 | 0.025796 |
| rs705702 | G | A | 1.95E-18 | 0.0785362 | 0.00896516 | 0.302645 | 287247 | 0.000267087 | 76.73979851 | 0.004946978 | -0.0555962 | 0.018348 |
| rs713427 | C | T | 8.38E-09 | 0.0571529 | 0.00992134 | 0.221764 | 287247 | 0.000115513 | 33.18431475 | 0.218653785 | 0.0305564 | 0.024999 |
| rs71641308 | T | C | 1.75E-09 | 0.0837373 | 0.0139108 | 0.0964292 | 287247 | 0.000126131 | 36.23520059 | 0.002430972 | 0.124634 | 0.044723 |
| rs72796365 | T | C | 4.49E-08 | 0.138961 | 0.0254015 | 0.0260409 | 287247 | 0.000104176 | 29.92706392 | 0.944830926 | 0.00468302 | 0.063691 |
| rs72866766 | C | T | 5.71E-09 | 0.0810729 | 0.0139181 | 0.0946304 | 287247 | 0.000118109 | 33.93036155 | 0.582197049 | -0.0186702 | 0.032221 |
| rs7321973 | A | C | 1.73E-19 | 0.0814066 | 0.00901603 | 0.295517 | 287247 | 0.000283733 | 81.52400613 | 0.54388615 | 0.0133525 | 0.02182 |
| rs76169968 | A | G | 2.85E-11 | -0.0943365 | 0.0141771 | 0.0989575 | 287247 | 0.000154121 | 44.27735703 | 0.995555005 | 0.000186983 | 0.032557 |
| rs76653830 | T | C | 2.20E-08 | 0.0754617 | 0.0134855 | 0.102668 | 287247 | 0.000108997 | 31.31237712 | 0.280792174 | 0.0487597 | 0.045427 |
| rs774121 | C | T | 1.35E-12 | -0.0673027 | 0.00949342 | 0.749377 | 287247 | 0.00017494 | 50.25931449 | 0.738375027 | 0.00708705 | 0.020635 |
| rs7844425 | G | T | 2.15E-09 | -0.0520926 | 0.00870196 | 0.355567 | 287247 | 0.000124741 | 35.8356164 | 0.887765983 | -0.00274577 | 0.019039 |
| rs7902146 | T | C | 3.71E-16 | -0.0774861 | 0.00951006 | 0.750339 | 287247 | 0.00023106 | 66.38623938 | 0.022843887 | 0.0475477 | 0.019515 |
| rs794999 | G | A | 1.02E-11 | -0.0666523 | 0.00979602 | 0.77136 | 287247 | 0.000161141 | 46.2943436 | 0.062383538 | 0.0398215 | 0.020109 |
| rs8006310 | G | A | 9.68E-11 | -0.0536927 | 0.00829636 | 0.504402 | 287247 | 0.000145793 | 41.88439873 | 0.890860083 | -0.0025322 | 0.01808 |
| rs8087237 | A | C | 6.54E-09 | -0.0482406 | 0.00831393 | 0.490285 | 287247 | 0.000117194 | 33.66740555 | 0.081217572 | 0.0317389 | 0.018458 |
| rs8193 | T | C | 4.41E-11 | 0.0570366 | 0.00865554 | 0.352657 | 287247 | 0.000151146 | 43.42260746 | 0.144377993 | 0.0273797 | 0.018929 |
| rs897586 | A | G | 1.87E-12 | -0.0630711 | 0.00895376 | 0.318214 | 287247 | 0.000172711 | 49.61887217 | 0.710300303 | 0.00711562 | 0.018936 |
| rs9292 | G | A | 1.18E-09 | -0.140954 | 0.0231718 | 0.968488 | 287247 | 0.000128802 | 37.00260018 | 0.90507694 | 0.00767941 | 0.060047 |
| rs9975418 | G | A | 1.06E-08 | 0.0613504 | 0.010725 | 0.179825 | 287247 | 0.000113903 | 32.72179894 | 0.284712805 | 0.0231959 | 0.021727 |

Abbreviations: A1, effect allele; A2, other allele; beta, effect size; se, standard error of beta; PVE, the proportion of variance explained by individual SNP. There are totally 85 valid instrumental SNP variables for hypothyroidism, with the PVE being 1.94%.The overall F statistic for the 85 SNPs is 66.80 (PVE=1.94%, k=85 and N=287247).

**Supplementary Table S3.** The instrumental SNP variables for hyperthyroidism on lung cancer

| rsid | A1 | A2 | hyperthyroidism | | | | | | | lung cancer | | |
| --- | --- | --- | --- | --- | --- | --- | --- | --- | --- | --- | --- | --- |
|  |  |  | P.x | beta.x | se.x | eaf | N | PVE | F | P.y | beta.y | se.y |
| rs11571297 | C | T | 5.55E-12 | -0.252377 | 0.0366258 | 0.376659 | 257552 | 0.000184323 | 47.48122724 | 0.183074981954095 | -0.0243921 | 0.017563 |
| rs179252 | G | T | 2.39E-17 | -0.296525 | 0.0349954 | 0.591492 | 257552 | 0.000278686 | 71.79551794 | 0.505291508598554 | -0.012022 | 0.017518 |
| rs2476601 | G | A | 5.44E-16 | -0.372343 | 0.0459614 | 0.859146 | 257552 | 0.000254756 | 65.62911029 | 0.971483142548622 | 0.0629654 | 0.028117 |
| rs73744913 | T | C | 1.07E-14 | 0.442377 | 0.0572245 | 0.0771435 | 257552 | 0.000231983 | 59.76097747 | 0.90492898719199 | -0.00388755 | 0.031416 |

Abbreviations:A1, effect allele; A2, other allele; beta, effect size; se, standard error of beta; PVE, the proportion of variance explained by individual SNP. There are totally 4 valid instrumental SNP variables for hyperthyroidism, with the PVE being0.095%. The overall F statistic for the 4 SNPs is 61.21 (PVE=0.095%, k=4 and N=257,552).

**Supplementary Table S4.** The instrumental SNP variables for TSH on lung cancer

| rsid | A1 | A2 | TSH | | | | | | | lung cancer | | |
| --- | --- | --- | --- | --- | --- | --- | --- | --- | --- | --- | --- | --- |
|  |  |  | P.x | beta.x | se.x | N | eaf | PVE | F | P.y | beta.y | se.y |
| rs1042673 | A | G | 3.57E-19 | -0.0546 | 0.0061 | 54288 | 0.4098 | 0.001473606 | 80.11422124 | 0.911912985 | 0.00202096 | 0.017985 |
| rs1045476 | A | G | 2.36E-09 | 0.049 | 0.0082 | 54288 | 0.3686 | 0.000657317 | 35.70659646 | 0.925678976 | -0.00225645 | 0.02368 |
| rs10748781 | A | C | 3.69E-13 | -0.0467 | 0.0064 | 54288 | 0.6877 | 0.000979815 | 53.24242321 | 0.430080746 | 0.0152598 | 0.01869 |
| rs1079418 | A | G | 8.23E-53 | 0.1009 | 0.0066 | 52424 | 0.7179 | 0.004438461 | 233.7103213 | 0.326042339 | 0.0194271 | 0.019783 |
| rs10814915 | T | C | 5.06E-12 | 0.0421 | 0.0061 | 54288 | 0.5417 | 0.000876637 | 47.63087083 | 0.648083553 | 0.00833968 | 0.017798 |
| rs10917469 | A | G | 3.95E-39 | 0.1112 | 0.0085 | 51018 | 0.8269 | 0.003343442 | 171.1412492 | 0.650535498 | 0.0112742 | 0.024563 |
| rs10957494 | A | G | 1.10E-09 | -0.0402 | 0.0066 | 54288 | 0.5911 | 0.00068291 | 37.0978068 | 0.532163397 | -0.0122042 | 0.018933 |
| rs11255790 | T | C | 6.83E-10 | -0.041 | 0.0066 | 54288 | 0.1937 | 0.000710342 | 38.58902826 | 0.616759716 | -0.0100746 | 0.01954 |
| rs1157994 | A | G | 5.28E-09 | -0.0904 | 0.0155 | 50232 | 0.03617 | 0.000676704 | 34.01387981 | 0.729916196 | 0.015595 | 0.042559 |
| rs11654194 | A | G | 1.82E-14 | 0.0467 | 0.0061 | 54288 | 0.2756 | 0.001078454 | 58.60816057 | 0.364702718 | 0.0165634 | 0.017655 |
| rs11732089 | T | C | 1.73E-51 | 0.115 | 0.0076 | 52047 | 0.8104 | 0.004379923 | 228.9558831 | 0.789007261 | 0.00625142 | 0.022979 |
| rs11755845 | T | C | 1.43E-25 | -0.0725 | 0.0069 | 54288 | 0.2001 | 0.002029512 | 110.3981591 | 0.122273966 | -0.03261 | 0.02001 |
| rs118039499 | A | C | 1.99E-14 | 0.1837 | 0.024 | 48736 | 0.992216 | 0.001200671 | 58.58386313 | 0.94435199 | 0.00559134 | 0.074578 |
| rs1203944 | T | C | 2.42E-12 | -0.0509 | 0.0073 | 54288 | 0.2775 | 0.000894741 | 48.61539788 | 0.214874065 | -0.0275811 | 0.02237 |
| rs12284404 | A | G | 2.48E-22 | -0.0667 | 0.0069 | 54288 | 0.1722 | 0.001718315 | 93.4410019 | 0.432438149 | -0.01604 | 0.019709 |
| rs12893151 | A | C | 1.02E-15 | -0.0624 | 0.0078 | 54288 | 0.1795 | 0.00117751 | 63.9976422 | 0.521714611 | 0.0147467 | 0.022838 |
| rs13015993 | A | G | 4.52E-32 | 0.0818 | 0.0069 | 52047 | 0.6016 | 0.002693032 | 140.5373425 | 0.717987693 | 0.00730361 | 0.019684 |
| rs13329353 | T | C | 5.17E-21 | 0.0614 | 0.0065 | 54288 | 0.658 | 0.001640941 | 89.22653521 | 0.620777546 | -0.00958681 | 0.018831 |
| rs143025968 | T | C | 2.63E-10 | 0.0835 | 0.0132 | 41566 | 0.07326 | 0.000961765 | 40.01328353 | 0.132332038 | 0.0507349 | 0.034321 |
| rs1663070 | T | C | 3.49E-11 | -0.0463 | 0.007 | 54288 | 0.6708 | 0.000805216 | 43.74716378 | 0.055853449 | 0.0409595 | 0.020134 |
| rs17020122 | T | C | 5.32E-20 | 0.1044 | 0.0114 | 51018 | 0.1424 | 0.001641174 | 83.86374827 | 0.666931147 | 0.0137747 | 0.031452 |
| rs17477923 | T | C | 2.57E-33 | 0.0826 | 0.0069 | 54288 | 0.8274 | 0.002632771 | 143.2999085 | 0.203311058 | -0.026308 | 0.019741 |
| rs17767491 | A | G | 3.35E-42 | 0.0883 | 0.0065 | 54288 | 0.7463 | 0.003387794 | 184.5349765 | 0.645291583 | 0.00894983 | 0.019248 |
| rs199452 | T | C | 1.29E-09 | 0.043 | 0.0071 | 54288 | 0.4107 | 0.000675185 | 36.67787903 | 0.605986571 | -0.0111631 | 0.020953 |
| rs2127387 | A | G | 1.10E-117 | 0.1435 | 0.0062 | 53074 | 0.6346 | 0.009992567 | 535.6783043 | 0.78766582 | 0.00504525 | 0.018485 |
| rs2272642 | A | G | 2.10E-08 | 0.0442 | 0.0079 | 54288 | 0.864 | 0.000576283 | 31.30216354 | 0.929708925 | 0.00210079 | 0.023321 |
| rs2284736 | A | G | 1.06E-11 | 0.0436 | 0.0064 | 54288 | 0.4528 | 0.000854158 | 46.40844647 | 0.366791278 | 0.0175725 | 0.018771 |
| rs2439301 | A | G | 8.15E-15 | -0.0587 | 0.0076 | 54288 | 0.2404 | 0.001097661 | 59.65310005 | 0.254031165 | -0.0260351 | 0.022912 |
| rs28502438 | T | C | 3.70E-08 | 0.0338 | 0.0061 | 54288 | 0.772 | 0.000565229 | 30.70136823 | 0.947777076 | -0.00121374 | 0.018183 |
| rs30227 | T | C | 7.59E-14 | -0.0468 | 0.0063 | 54288 | 0.6245 | 0.001015466 | 55.18164047 | 0.982957 | 0.000404082 | 0.018574 |
| rs334725 | A | G | 2.45E-32 | 0.1737 | 0.0147 | 51018 | 0.92399 | 0.002729321 | 139.6200991 | 0.005617978 | -0.127866 | 0.050152 |
| rs398745 | A | C | 3.97E-17 | -0.052 | 0.0062 | 54288 | 0.5192 | 0.001294068 | 70.34080081 | 0.587509644 | -0.0100453 | 0.018003 |
| rs4445669 | T | C | 5.76E-11 | -0.0397 | 0.0061 | 54288 | 0.6213 | 0.000779612 | 42.35506412 | 0.008698012 | 0.0479819 | 0.017118 |
| rs4804413 | T | C | 8.64E-18 | 0.0532 | 0.0062 | 51942 | 0.3654 | 0.001415488 | 73.6246364 | 0.626241725 | 0.00892604 | 0.018164 |
| rs4933466 | A | G | 5.13E-10 | 0.0395 | 0.0063 | 54288 | 0.5513 | 0.000723594 | 39.30946131 | 0.215973765 | -0.0230189 | 0.017854 |
| rs544873 | A | G | 2.53E-15 | 0.05 | 0.0063 | 54288 | 0.3897 | 0.001158915 | 62.98583771 | 0.75861775 | -0.00568915 | 0.018078 |
| rs59381142 | A | G | 1.70E-14 | -0.058 | 0.0076 | 52048 | 0.2184 | 0.001117736 | 58.23875926 | 0.582270785 | -0.01211 | 0.021287 |
| rs7329958 | T | C | 1.13E-11 | -0.0439 | 0.0065 | 54288 | 0.2683 | 0.000839525 | 45.61275741 | 0.535980513 | 0.012599 | 0.019706 |
| rs7529705 | A | G | 1.39E-16 | 0.0531 | 0.0064 | 51018 | 0.3439 | 0.001347473 | 68.83543618 | 0.273582932 | -0.0203731 | 0.017905 |
| rs8015085 | A | G | 2.45E-18 | 0.0671 | 0.0077 | 54288 | 0.2097 | 0.001396859 | 75.93597788 | 0.610687457 | -0.0115211 | 0.021882 |
| rs9298749 | A | C | 8.80E-10 | -0.0393 | 0.0064 | 54288 | 0.473 | 0.000694096 | 37.70588623 | 0.846159043 | 0.00372406 | 0.018909 |
| rs9497965 | T | C | 9.81E-13 | 0.0444 | 0.0062 | 54288 | 0.3782 | 0.000943775 | 51.28218975 | 0.965779094 | -0.000794315 | 0.018173 |

Abbreviations: A1, effect allele; A2, other allele; TSH, thyroid stimulating hormone; beta, effect size; se, standard error of beta; PVE, the proportion of variance explained by individual SNP. There are totally 42 valid instrumental SNP variables for TSH, with the PVE being 6.93%.The overall F statistic for the 42 SNPs is 96.17(PVE=6.93%, k=42).

**Supplementary Table S5.** The instrumental SNP variables for FT4 on lung cancer

| rsid | A1 | A2 | FT4 | | | | | | lung cancer | | |
| --- | --- | --- | --- | --- | --- | --- | --- | --- | --- | --- | --- |
|  |  |  | P.x | beta.x | se.x | N | PVE | F | P.y | beta.y | se.y |
| rs10119187 | T | C | 4.11E-09 | 0.0497 | 0.0085 | 49269 | 0.000693426 | 34.18670907 | 0.813714959 | 0.00556051 | 0.023191 |
| rs10739496 | T | C | 4.20E-30 | 0.0777 | 0.0068 | 49269 | 0.002643024 | 130.55893 | 0.525201551 | 0.0121859 | 0.018595 |
| rs10818937 | T | C | 1.31E-11 | -0.0475 | 0.007 | 49269 | 0.000933709 | 46.0440492 | 0.489504851 | 0.0134442 | 0.019345 |
| rs10946313 | T | C | 2.28E-11 | 0.0455 | 0.0068 | 49269 | 0.000907897 | 44.77002512 | 0.300861766 | -0.0197712 | 0.01839 |
| rs11039355 | T | C | 3.51E-08 | -0.0385 | 0.007 | 49269 | 0.0006136 | 30.24877205 | 0.79576109 | 0.0049965 | 0.019036 |
| rs113107469 | T | C | 1.00E-19 | 0.1996 | 0.022 | 49269 | 0.001667927 | 82.31103874 | 0.968925978 | 0.00246895 | 0.059773 |
| rs17185536 | T | C | 1.93E-19 | 0.0726 | 0.0081 | 49269 | 0.001627878 | 80.33144401 | 0.6441485 | -0.00993014 | 0.020841 |
| rs2235544 | A | C | 4.20E-101 | 0.1387 | 0.0065 | 49269 | 0.009157086 | 455.3114574 | 0.812521946 | -0.00430173 | 0.017896 |
| rs225014 | T | C | 1.83E-15 | 0.0535 | 0.0067 | 49269 | 0.001292476 | 63.7588285 | 0.670183915 | 0.00811102 | 0.018846 |
| rs35799415 | T | C | 1.28E-12 | 0.0485 | 0.0068 | 49269 | 0.001031439 | 50.86839347 | 0.305231956 | -0.0196428 | 0.019177 |
| rs4149056 | T | C | 1.34E-08 | -0.0506 | 0.0089 | 49269 | 0.000655635 | 32.32238437 | 0.466212593 | -0.0175949 | 0.023173 |
| rs4842131 | T | C | 7.68E-44 | -0.1037 | 0.0075 | 44811 | 0.004248166 | 191.1681785 | 0.704130246 | 0.00744162 | 0.019081 |
| rs4954192 | T | C | 8.38E-09 | -0.0409 | 0.0071 | 44902 | 0.000738488 | 33.18261239 | 0.770489988 | -0.00586818 | 0.019606 |
| rs56069042 | A | G | 1.16E-08 | 0.1061 | 0.0186 | 49269 | 0.000660001 | 32.53772988 | 0.219773842 | 0.0656838 | 0.054266 |
| rs6785807 | A | G | 2.47E-10 | -0.059 | 0.0093 | 49269 | 0.000816225 | 40.24579367 | 0.16033303 | 0.0347347 | 0.025005 |
| rs6855450 | T | C | 2.76E-24 | 0.1164 | 0.0115 | 47314 | 0.002160634 | 102.4452724 | 0.326312717 | -0.0332218 | 0.031678 |
| rs72783371 | A | C | 2.77E-08 | 0.0671 | 0.0121 | 49269 | 0.000623777 | 30.75081778 | 0.047103155 | -0.0652481 | 0.029816 |
| rs73405691 | A | G | 1.61E-08 | 0.0556 | 0.0098 | 49269 | 0.00065289 | 32.18694826 | 0.635330932 | 0.0132656 | 0.027581 |
| rs9356988 | A | G | 3.56E-12 | -0.051 | 0.0073 | 49269 | 0.000989671 | 48.80642553 | 0.086272027 | -0.0346473 | 0.019129 |
| rs951366 | T | C | 4.39E-08 | 0.0367 | 0.0067 | 49269 | 0.000608617 | 30.00301459 | 0.647339339 | 0.00860487 | 0.018626 |

Abbreviations: A1, effect allele; A2, other allele; FT4, free thyroxine; beta, effect size; se, standard error of beta; PVE, the proportion of variance explained by individual SNP. There are totally 20 valid instrumental SNP variables for FT4, with the PVE being 3.27%.The overall F statistic for the 20 SNPs is 83.30(PVE=3.27%, k=20).

**Supplementary Table S6.** The instrumental SNP variables for lung cancer on hypothyroidism

| rsid | A1 | A2 | lung cancer | | | | | | | hypothyroidism | | |
| --- | --- | --- | --- | --- | --- | --- | --- | --- | --- | --- | --- | --- |
|  |  |  | P.x | beta.x | se.x | eaf | N | PVE | F | P.y | beta.y | se.y |
| rs11571818 | C | T | 2.63E-12 | 0.660847 | 0.16686 | 0.0113361 | 27209 | 0.000576149 | 15.68431092 | 0.216883 | -0.0531529 | 0.0430437 |
| rs37004 | T | C | 3.19E-13 | -0.17209 | 0.019417 | 0.226187 | 27209 | 0.002878608 | 78.54437422 | 0.320599 | 0.00993898 | 0.0100067 |
| rs446975 | T | G | 6.42E-18 | -0.261532 | 0.022649 | 0.115137 | 27209 | 0.004876587 | 133.3274836 | 0.862944 | -0.00211443 | 0.0122485 |

Abbreviations: A1, effect allele; A2, other allele; beta, effect size; se, standard error of beta; PVE, the proportion of variance explained by individual SNP. There are totally 3 valid instrumental SNP variables for lung cancer, with the PVE being 0.83%.The overall F statistic for the 3 SNPs is 76.19 (PVE=0.83%, k=3 and N=27209).

**Supplementary Table S7.** The instrumental SNP variables for lung cancer on hyperthyroidism

| rsid | A1 | A2 | lung cancer | | | | | | hyperthyroidism | | |
| --- | --- | --- | --- | --- | --- | --- | --- | --- | --- | --- | --- |
|  |  |  | P.x | beta.x | se.x | N | PVE | F | P.y | beta.y | se.y |
| rs11571818 | C | T | 2.63E-12 | 0.660847 | 0.16686 | 27209 | 0.000576149 | 15.68431092 | 0.0690526 | -0.337234 | 0.185489 |
| rs37004 | T | C | 3.19E-13 | -0.17209 | 0.019417 | 27209 | 0.002878608 | 78.54437422 | 0.578589 | 0.0237325 | 0.0427268 |
| rs446975 | T | G | 6.42E-18 | -0.261532 | 0.022649 | 27209 | 0.004876587 | 133.3274836 | 0.296503 | 0.0543601 | 0.052071 |
| rs8040868 | C | T | 4.97E-60 | 0.301974 | 0.024535 | 27209 | 0.005536601 | 151.47294 | 0.00836778 | 0.0946655 | 0.0359009 |

Abbreviations: A1, effect allele; A2, other allele; beta, effect size; se, standard error of beta; PVE, the proportion of variance explained by individual SNP. There are totally 4 valid instrumental SNP variables for lung cancer, with the PVE being 1.39%.The overall F statistic for the 4 SNPs is 95.64 (PVE=1.39%, k=4 and N=27209).

**Supplementary Table S8.** The instrumental SNP variables for lung cancer on TSH

| rsid | A1 | A2 | lung cancer | | | | | | TSH | | |
| --- | --- | --- | --- | --- | --- | --- | --- | --- | --- | --- | --- |
|  |  |  | P.x | beta.x | se.x | N | PVE | F | P.y | beta.y | se.y |
| rs37004 | T | C | 3.19E-13 | -0.17209 | 0.019417 | 27209 | 0.002878608 | 78.54437422 | 0.03066 | -0.0175 | 0.0081 |
| rs446975 | T | G | 6.42E-18 | -0.261532 | 0.022649 | 27209 | 0.004876587 | 133.3274836 | 0.7708 | -0.0027 | 0.0091 |
| rs501942 | T | C | 1.47E-10 | 0.186841 | 0.034137 | 27209 | 0.001099773 | 29.95447676 | 0.1427 | -0.0162 | 0.011 |
| rs8040868 | C | T | 4.97E-60 | 0.301974 | 0.024535 | 27209 | 0.005536601 | 151.47294 | 0.2927 | -0.0065 | 0.0062 |

Abbreviations: A1, effect allele; A2, other allele; beta, effect size; se, standard error of beta; PVE, the proportion of variance explained by individual SNP. There are totally 4 valid instrumental SNP variables for lung cancer, with the PVE being 1.44%.The overall F statistic for the 4 SNPs is 99.31 (PVE=1.44%, k=4 and N=27209).

**Supplementary Table S9.** The instrumental SNP variables for lung cancer on FT4

| rsid | A1 | A2 | lung cancer | | | | | | FT4 | | |
| --- | --- | --- | --- | --- | --- | --- | --- | --- | --- | --- | --- |
|  |  |  | P.x | beta.x | se.x | N | PVE | F | P.y | beta.y | se.y |
| rs11571818 | C | T | 2.63E-12 | 0.660847 | 0.16686 | 27209 | 0.000576149 | 15.68431092 | 0.1262 | 0.0632 | 0.0413 |
| rs37004 | T | C | 3.19E-13 | -0.17209 | 0.019417 | 27209 | 0.002878608 | 78.54437422 | 0.2933 | -0.009 | 0.0086 |
| rs446975 | T | G | 6.42E-18 | -0.261532 | 0.022649 | 27209 | 0.004876587 | 133.3274836 | 0.7174 | -0.0035 | 0.0098 |
| rs501942 | T | C | 1.47E-10 | 0.186841 | 0.034137 | 27209 | 0.001099773 | 29.95447676 | 0.0001238 | 0.0448 | 0.0117 |
| rs8040868 | C | T | 4.97E-60 | 0.301974 | 0.024535 | 27209 | 0.005536601 | 151.47294 | 0.9199 | 7.00E-04 | 0.0067 |

Abbreviations: A1, effect allele; A2 other allele; beta, effect size; se, standard error of beta; PVE, the proportion of variance explained by individual SNP. There are totally 5 valid instrumental SNP variables for lung cancer, with the PVE being 1.50%.The overall F statistic for the 5 SNPs is 82.67 (PVE=1.50%, k=5 and N=27209).

**Supplementary Table S10.** The instrumental SNP variables for hypothyroidism on lung adenocarcinoma

| rsid | A1 | A2 | hypothyroidism | | | | | | | lung adenocarcinoma | | |
| --- | --- | --- | --- | --- | --- | --- | --- | --- | --- | --- | --- | --- |
|  |  |  | P.x | beta.x | se.x | eaf | N | PVE | F | P.y | se.y | beta.y |
| rs10008892 | A | C | 1.06E-30 | -0.151395 | 0.0131437 | 0.123091 | 287247 | 0.00046167 | 132.6736768 | 0.277457839 | 0.035391 | 0.0382837 |
| rs10112995 | A | G | 1.32E-08 | 0.0483905 | 0.00851337 | 0.612318 | 287247 | 0.000112464 | 32.30830528 | 0.829212041 | 0.028531 | 0.00637427 |
| rs10118880 | A | G | 4.57E-13 | -0.0666212 | 0.00920486 | 0.723956 | 287247 | 0.000182329 | 52.38265457 | 0.075340761 | 0.031715 | -0.0550183 |
| rs10166287 | G | T | 2.21E-10 | -0.0881637 | 0.0138928 | 0.102756 | 287247 | 0.000140179 | 40.27142756 | 0.268264991 | 0.037524 | -0.0452244 |
| rs10259879 | G | A | 6.27E-09 | 0.0571615 | 0.00983933 | 0.232158 | 287247 | 0.000117482 | 33.74995177 | 0.810391916 | 0.033039 | -0.00826506 |
| rs10481413 | T | C | 1.28E-09 | -0.050671 | 0.008348 | 0.444792 | 287247 | 0.000128246 | 36.84266574 | 0.467208785 | 0.028352 | -0.02076 |
| rs10494077 | A | G | 2.27E-28 | 0.110234 | 0.00997883 | 0.213359 | 287247 | 0.000424651 | 122.0306323 | 0.899978014 | 0.033085 | 0.00427983 |
| rs10748781 | A | C | 2.88E-20 | -0.0801497 | 0.00869 | 0.652669 | 287247 | 0.00029606 | 85.06702123 | 0.233608155 | 0.028087 | 0.0356976 |
| rs1079418 | G | A | 2.66E-19 | -0.0852228 | 0.00948814 | 0.261894 | 287247 | 0.000280784 | 80.67638048 | 0.12692115 | 0.030588 | 0.0459194 |
| rs10814915 | C | T | 3.58E-17 | -0.0698807 | 0.00829364 | 0.543644 | 287247 | 0.000247093 | 70.99391223 | 0.627357364 | 0.027227 | 0.0137775 |
| rs10858026 | G | T | 4.07E-10 | 0.0666008 | 0.010654 | 0.807372 | 287247 | 0.000136025 | 39.07783394 | 0.695166764 | 0.032975 | -0.0131778 |
| rs10948107 | A | G | 1.07E-10 | -0.0672386 | 0.0104134 | 0.202244 | 287247 | 0.000145122 | 41.69166111 | 0.496834791 | 0.031059 | -0.0222814 |
| rs11079035 | A | G | 4.69E-13 | 0.0738873 | 0.0102138 | 0.202278 | 287247 | 0.00018215 | 52.33134184 | 0.853267099 | 0.03576 | -0.00690579 |
| rs111352680 | A | G | 1.23E-08 | -0.0509487 | 0.00894548 | 0.323629 | 287247 | 0.000112916 | 32.43813614 | 0.027118771 | 0.034583 | 0.0733629 |
| rs11571297 | C | T | 4.06E-45 | -0.121325 | 0.0086075 | 0.373405 | 287247 | 0.000691179 | 198.6751334 | 0.535751012 | 0.027026 | -0.0175094 |
| rs116776245 | A | G | 3.54E-09 | 0.0869905 | 0.014733 | 0.0833056 | 287247 | 0.000121354 | 34.86247795 | 0.091873445 | 0.054808 | -0.109835 |
| rs11683207 | C | T | 8.36E-09 | -0.0536415 | 0.0093111 | 0.278037 | 287247 | 0.00011553 | 33.18920286 | 0.718234066 | 0.037462 | 0.0138388 |
| rs11969311 | C | A | 1.22E-08 | -0.0545027 | 0.00956667 | 0.252339 | 287247 | 0.000112982 | 32.45722783 | 0.23325878 | 0.032775 | -0.0421792 |
| rs1203943 | C | T | 1.83E-23 | 0.103531 | 0.010372 | 0.79178 | 287247 | 0.000346745 | 99.63519559 | 0.457485149 | 0.033188 | -0.0248506 |
| rs12091047 | T | C | 1.08E-25 | 0.0927867 | 0.00885449 | 0.316692 | 287247 | 0.00038214 | 109.8098582 | 0.564441581 | 0.029173 | 0.0170115 |
| rs12129508 | A | G | 4.49E-08 | 0.0690302 | 0.0126184 | 0.120108 | 287247 | 0.000104176 | 29.92723948 | 0.468676308 | 0.039883 | 0.0291927 |
| rs12206423 | T | C | 1.24E-08 | 0.0655998 | 0.0115195 | 0.14809 | 287247 | 0.000112884 | 32.429089 | 0.11152494 | 0.042972 | 0.0666366 |
| rs123378 | A | G | 3.85E-09 | -0.0502193 | 0.00852516 | 0.617779 | 287247 | 0.000120789 | 34.70029834 | 0.069877925 | 0.0282 | 0.0556903 |
| rs12506688 | T | C | 5.20E-14 | 0.0687936 | 0.00913992 | 0.281399 | 287247 | 0.000197183 | 56.65108878 | 0.421037715 | 0.030626 | 0.0247716 |
| rs12697352 | A | G | 1.49E-09 | -0.0514445 | 0.00850986 | 0.395739 | 287247 | 0.00012721 | 36.54517506 | 0.88839192 | 0.030489 | 0.00439134 |
| rs12736474 | A | G | 2.35E-09 | 0.053367 | 0.0089364 | 0.31216 | 287247 | 0.00012414 | 35.6629575 | 0.367949316 | 0.028752 | -0.0274061 |
| rs12756019 | A | G | 2.38E-11 | -0.0566832 | 0.00848487 | 0.60567 | 287247 | 0.000155344 | 44.6288104 | 0.217905905 | 0.030034 | -0.0367214 |
| rs13137589 | G | A | 4.42E-13 | -0.0627853 | 0.00866943 | 0.368031 | 287247 | 0.000182558 | 52.4483506 | 0.994575004 | 0.028608 | -0.00020002 |
| rs1317983 | C | T | 1.25E-27 | 0.0980504 | 0.00900134 | 0.680676 | 287247 | 0.000412904 | 118.6537267 | 0.83165308 | 0.031027 | -0.00675712 |
| rs13220783 | T | G | 3.99E-09 | 0.0499938 | 0.00849568 | 0.380325 | 287247 | 0.000120539 | 34.62844433 | 0.884901029 | 0.028318 | 0.00419818 |
| rs13447704 | C | T | 2.09E-08 | -0.14695 | 0.0262205 | 0.0268588 | 287247 | 0.000109334 | 31.40900791 | 0.687522634 | 0.093533 | -0.0436259 |
| rs1371867 | C | A | 5.71E-09 | 0.0487005 | 0.00836052 | 0.428582 | 287247 | 0.000118112 | 33.93104507 | 0.864404064 | 0.027541 | -0.00485878 |
| rs141686764 | G | A | 9.11E-09 | -0.182081 | 0.0316857 | 0.0187788 | 287247 | 0.000114947 | 33.02171493 | 0.851003997 | 0.096924 | 0.0197507 |
| rs1441172 | T | C | 3.43E-09 | -0.0533644 | 0.00903023 | 0.313428 | 287247 | 0.000121562 | 34.92228191 | 0.638616301 | 0.02859 | 0.0136257 |
| rs1534430 | T | C | 2.76E-11 | -0.056034 | 0.00841488 | 0.417775 | 287247 | 0.000154342 | 44.34088669 | 0.473424795 | 0.028538 | 0.0206121 |
| rs1788105 | G | A | 2.77E-08 | -0.0462256 | 0.00832056 | 0.547645 | 287247 | 0.000107438 | 30.86433873 | 0.905803929 | 0.027404 | -0.00332148 |
| rs2104047 | C | T | 1.20E-08 | 0.0514534 | 0.00902662 | 0.693491 | 287247 | 0.000113103 | 32.49187785 | 0.520628574 | 0.030199 | -0.0195977 |
| rs2111485 | G | A | 5.67E-18 | 0.0727979 | 0.00842671 | 0.583695 | 287247 | 0.000259749 | 74.63088343 | 0.436749081 | 0.028895 | -0.0226007 |
| rs2235544 | A | C | 4.66E-10 | -0.0519065 | 0.0083313 | 0.5448 | 287247 | 0.000135115 | 38.81637023 | 0.098946401 | 0.025956 | 0.0460858 |
| rs229528 | T | C | 6.96E-21 | 0.0790766 | 0.0084354 | 0.396388 | 287247 | 0.000305841 | 87.87828838 | 0.048963223 | 0.026128 | -0.0559356 |
| rs2402240 | A | G | 1.92E-18 | 0.0940805 | 0.0107375 | 0.176345 | 287247 | 0.000267191 | 76.76970224 | 0.379238133 | 0.03323 | -0.0312059 |
| rs244689 | G | A | 3.29E-14 | -0.0718721 | 0.00947387 | 0.749122 | 287247 | 0.00020032 | 57.55231573 | 0.407944423 | 0.037486 | 0.0333562 |
| rs2476601 | G | A | 1.71E-162 | -0.304028 | 0.0111921 | 0.854687 | 287247 | 0.002562325 | 737.9058911 | 0.124517101 | 0.043486 | 0.0755687 |
| rs2531989 | A | G | 5.90E-11 | -0.0695526 | 0.010625 | 0.817382 | 287247 | 0.000149159 | 42.85148111 | 0.752178646 | 0.036333 | 0.0120523 |
| rs2680296 | A | C | 1.17E-08 | 0.0473154 | 0.00829442 | 0.468365 | 287247 | 0.000113273 | 32.54093014 | 0.928015009 | 0.027815 | 0.00257668 |
| rs28391281 | C | T | 3.99E-09 | -0.0488629 | 0.00830364 | 0.546421 | 287247 | 0.000120535 | 34.627284 | 0.275007159 | 0.030013 | -0.0350056 |
| rs28582094 | G | A | 3.81E-09 | 0.0552873 | 0.00938328 | 0.261193 | 287247 | 0.000120846 | 34.71669602 | 0.894922032 | 0.031571 | -0.00432233 |
| rs2928167 | G | A | 3.52E-22 | -0.101973 | 0.0105297 | 0.199071 | 287247 | 0.000326393 | 93.78542973 | 0.134022897 | 0.043106 | 0.0631748 |
| rs2958154 | T | C | 7.35E-12 | -0.0616386 | 0.0089973 | 0.699391 | 287247 | 0.000163363 | 46.93297725 | 0.783276325 | 0.02899 | -0.00814077 |
| rs30233 | A | G | 4.58E-12 | -0.0582169 | 0.00841513 | 0.586872 | 287247 | 0.00016659 | 47.86008537 | 0.50250918 | 0.02747 | 0.0193153 |
| rs35717611 | T | C | 1.26E-08 | 0.0487793 | 0.0085697 | 0.378643 | 287247 | 0.000112781 | 32.39939242 | 0.806949023 | 0.032315 | -0.00822675 |
| rs3946137 | G | A | 1.22E-09 | 0.0526375 | 0.00866007 | 0.349188 | 287247 | 0.000128599 | 36.94406503 | 0.958253027 | 0.030714 | -0.00166038 |
| rs41177 | A | G | 2.48E-09 | -0.0504861 | 0.00846668 | 0.404374 | 287247 | 0.000123768 | 35.55611735 | 0.13420015 | 0.029355 | 0.0433056 |
| rs4409785 | C | T | 9.15E-16 | 0.0882346 | 0.0109776 | 0.165925 | 287247 | 0.000224859 | 64.60409017 | 0.55169875 | 0.036055 | -0.0227907 |
| rs4575545 | A | G | 1.12E-20 | -0.0852985 | 0.00914861 | 0.302816 | 287247 | 0.000302542 | 86.92997119 | 0.298686779 | 0.031053 | 0.0322077 |
| rs4606850 | T | C | 2.55E-08 | 0.0616895 | 0.0110754 | 0.165369 | 287247 | 0.000107994 | 31.0242032 | 0.940767921 | 0.035258 | -0.0027217 |
| rs4704397 | A | G | 8.89E-56 | 0.131555 | 0.00836138 | 0.41002 | 287247 | 0.000861052 | 247.5459192 | 0.927771014 | 0.028483 | 0.00264849 |
| rs4804433 | T | G | 2.49E-17 | -0.0754197 | 0.00890613 | 0.692424 | 287247 | 0.00024959 | 71.71145216 | 0.256274857 | 0.034424 | -0.0388639 |
| rs4853459 | C | T | 8.01E-43 | -0.132855 | 0.0096853 | 0.769419 | 287247 | 0.000654621 | 188.1597073 | 0.369412001 | 0.031629 | 0.0302376 |
| rs56983610 | A | C | 5.30E-18 | -0.108696 | 0.0125708 | 0.13032 | 287247 | 0.000260215 | 74.76498787 | 0.286988173 | 0.037534 | 0.0398282 |
| rs57652885 | T | C | 2.65E-09 | -0.112845 | 0.0189593 | 0.0530945 | 287247 | 0.000123314 | 35.42558418 | 0.273171885 | 0.092383 | -0.128953 |
| rs61759532 | T | C | 1.20E-12 | 0.0747524 | 0.0105202 | 0.188962 | 287247 | 0.00017574 | 50.48929016 | 0.072356908 | 0.036241 | -0.0728643 |
| rs61916675 | G | A | 3.01E-11 | 0.0590178 | 0.00888003 | 0.316646 | 287247 | 0.00015375 | 44.17068586 | 0.969938951 | 0.029522 | -0.00114666 |
| rs6724363 | T | G | 7.67E-24 | 0.0864868 | 0.00859042 | 0.624805 | 287247 | 0.000352746 | 101.3602885 | 0.953790956 | 0.03063 | -0.00182633 |
| rs6831973 | C | T | 4.47E-12 | 0.0577679 | 0.00834607 | 0.555424 | 287247 | 0.000166756 | 47.90778488 | 0.110364907 | 0.026457 | 0.0454473 |
| rs6914622 | T | G | 4.25E-14 | 0.0716868 | 0.00949102 | 0.254521 | 287247 | 0.000198569 | 57.04920385 | 0.573710766 | 0.029402 | -0.0173456 |
| rs7043516 | C | A | 1.45E-18 | -0.0998544 | 0.0113558 | 0.166059 | 287247 | 0.000269108 | 77.32072524 | 0.940945999 | 0.039343 | 0.00302243 |
| rs705702 | G | A | 1.95E-18 | 0.0785362 | 0.00896516 | 0.302645 | 287247 | 0.000267087 | 76.73979851 | 0.632553117 | 0.029176 | -0.0145828 |
| rs713427 | C | T | 8.38E-09 | 0.0571529 | 0.00992134 | 0.221764 | 287247 | 0.000115513 | 33.18431475 | 0.028168259 | 0.041104 | 0.0860171 |
| rs71641308 | T | C | 1.75E-09 | 0.0837373 | 0.0139108 | 0.0964292 | 287247 | 0.000126131 | 36.23520059 | 0.003884991 | 0.073418 | 0.187253 |
| rs72796365 | T | C | 4.49E-08 | 0.138961 | 0.0254015 | 0.0260409 | 287247 | 0.000104176 | 29.92706392 | 0.184318106 | 0.085184 | -0.145295 |
| rs72866766 | C | T | 5.71E-09 | 0.0810729 | 0.0139181 | 0.0946304 | 287247 | 0.000118109 | 33.93036155 | 0.092337901 | 0.045143 | -0.0871867 |
| rs7321973 | A | C | 1.73E-19 | 0.0814066 | 0.00901603 | 0.295517 | 287247 | 0.000283733 | 81.52400613 | 0.344772803 | 0.033655 | 0.0318281 |
| rs76169968 | A | G | 2.85E-11 | -0.0943365 | 0.0141771 | 0.0989575 | 287247 | 0.000154121 | 44.27735703 | 0.663677356 | 0.048693 | -0.0227948 |
| rs76653830 | T | C | 2.20E-08 | 0.0754617 | 0.0134855 | 0.102668 | 287247 | 0.000108997 | 31.31237712 | 0.764712167 | 0.06553 | -0.0214821 |
| rs774121 | C | T | 1.35E-12 | -0.0673027 | 0.00949342 | 0.749377 | 287247 | 0.00017494 | 50.25931449 | 0.860515901 | 0.031487 | 0.00574447 |
| rs7844425 | G | T | 2.15E-09 | -0.0520926 | 0.00870196 | 0.355567 | 287247 | 0.000124741 | 35.8356164 | 0.858826032 | 0.029234 | -0.00538346 |
| rs7902146 | T | C | 3.71E-16 | -0.0774861 | 0.00951006 | 0.750339 | 287247 | 0.00023106 | 66.38623938 | 0.699582601 | 0.030722 | 0.0123844 |
| rs794999 | G | A | 1.02E-11 | -0.0666523 | 0.00979602 | 0.77136 | 287247 | 0.000161141 | 46.2943436 | 0.157931951 | 0.030612 | 0.0467803 |
| rs8006310 | G | A | 9.68E-11 | -0.0536927 | 0.00829636 | 0.504402 | 287247 | 0.000145793 | 41.88439873 | 0.431950519 | 0.028598 | 0.0225968 |
| rs8087237 | A | C | 6.54E-09 | -0.0482406 | 0.00831393 | 0.490285 | 287247 | 0.000117194 | 33.66740555 | 0.101322991 | 0.028779 | 0.0462784 |
| rs8193 | T | C | 4.41E-11 | 0.0570366 | 0.00865554 | 0.352657 | 287247 | 0.000151146 | 43.42260746 | 0.286518059 | 0.028926 | 0.0307484 |
| rs897586 | A | G | 1.87E-12 | -0.0630711 | 0.00895376 | 0.318214 | 287247 | 0.000172711 | 49.61887217 | 0.942208027 | 0.02877 | -0.00215131 |
| rs9292 | G | A | 1.18E-09 | -0.140954 | 0.0231718 | 0.968488 | 287247 | 0.000128802 | 37.00260018 | 0.859294041 | 0.093713 | 0.0187395 |
| rs9975418 | G | A | 1.06E-08 | 0.0613504 | 0.010725 | 0.179825 | 287247 | 0.000113903 | 32.72179894 | 0.015840905 | 0.034809 | 0.0800802 |

Abbreviations: A1, effect allele; A2, other allele; beta, effect size; se, standard error of beta; PVE, the proportion of variance explained by individual SNP. There are totally 85 valid instrumental SNP variables for hypothyroidism, with the PVE being 1.94%.The overall F statistic for the 85 SNPs is 66.80 (PVE=1.94%, k=85 and N=287247).

**Supplementary Table S11.** The instrumental SNP variables for hypothyroidism on squamous cell lung cancer

| rsid | A1 | A2 | hypothyroidism | | | | | | | squamous cell lung cancer | | |
| --- | --- | --- | --- | --- | --- | --- | --- | --- | --- | --- | --- | --- |
|  |  |  | P.x | beta.x | se.x | eaf | N | PVE | F | se.y | beta.y | P.y |
| rs10008892 | A | C | 1.06E-30 | -0.151395 | 0.0131437 | 0.123091 | 287247 | 0.00046167 | 132.6736768 | 0.034441 | -0.0202129 | 0.57885928 |
| rs10112995 | A | G | 1.32E-08 | 0.0483905 | 0.00851337 | 0.612318 | 287247 | 0.000112464 | 32.30830528 | 0.027739 | 0.0372503 | 0.208750248 |
| rs10118880 | A | G | 4.57E-13 | -0.0666212 | 0.00920486 | 0.723956 | 287247 | 0.000182329 | 52.38265457 | 0.031656 | -0.0546965 | 0.07659669 |
| rs10166287 | G | T | 2.21E-10 | -0.0881637 | 0.0138928 | 0.102756 | 287247 | 0.000140179 | 40.27142756 | 0.043206 | 0.0748941 | 0.072851752 |
| rs10259879 | G | A | 6.27E-09 | 0.0571615 | 0.00983933 | 0.232158 | 287247 | 0.000117482 | 33.74995177 | 0.033476 | 0.00477757 | 0.889700019 |
| rs10481413 | T | C | 1.28E-09 | -0.050671 | 0.008348 | 0.444792 | 287247 | 0.000128246 | 36.84266574 | 0.027782 | 0.00540759 | 0.850667028 |
| rs10494077 | A | G | 2.27E-28 | 0.110234 | 0.00997883 | 0.213359 | 287247 | 0.000424651 | 122.0306323 | 0.033493 | 0.015128 | 0.657374722 |
| rs10748781 | A | C | 2.88E-20 | -0.0801497 | 0.00869 | 0.652669 | 287247 | 0.00029606 | 85.06702123 | 0.028951 | 0.00461764 | 0.877434083 |
| rs1079418 | G | A | 2.66E-19 | -0.0852228 | 0.00948814 | 0.261894 | 287247 | 0.000280784 | 80.67638048 | 0.029374 | -0.0153258 | 0.618047708 |
| rs10814915 | C | T | 3.58E-17 | -0.0698807 | 0.00829364 | 0.543644 | 287247 | 0.000247093 | 70.99391223 | 0.025655 | 0.0743346 | 0.008905945 |
| rs10858026 | G | T | 4.07E-10 | 0.0666008 | 0.010654 | 0.807372 | 287247 | 0.000136025 | 39.07783394 | 0.033597 | -0.0177584 | 0.602785782 |
| rs10948107 | A | G | 1.07E-10 | -0.0672386 | 0.0104134 | 0.202244 | 287247 | 0.000145122 | 41.69166111 | 0.03185 | 0.00122924 | 0.97013305 |
| rs11079035 | A | G | 4.69E-13 | 0.0738873 | 0.0102138 | 0.202278 | 287247 | 0.00018215 | 52.33134184 | 0.037267 | 0.029884 | 0.425704264 |
| rs111352680 | A | G | 1.23E-08 | -0.0509487 | 0.00894548 | 0.323629 | 287247 | 0.000112916 | 32.43813614 | 0.032517 | 0.016205 | 0.623845433 |
| rs11571297 | C | T | 4.06E-45 | -0.121325 | 0.0086075 | 0.373405 | 287247 | 0.000691179 | 198.6751334 | 0.026813 | -0.0340802 | 0.232173153 |
| rs116776245 | A | G | 3.54E-09 | 0.0869905 | 0.014733 | 0.0833056 | 287247 | 0.000121354 | 34.86247795 | 0.054127 | -0.0940305 | 0.136932015 |
| rs11683207 | C | T | 8.36E-09 | -0.0536415 | 0.0093111 | 0.278037 | 287247 | 0.00011553 | 33.18920286 | 0.036149 | -0.02185 | 0.568905327 |
| rs11969311 | C | A | 1.22E-08 | -0.0545027 | 0.00956667 | 0.252339 | 287247 | 0.000112982 | 32.45722783 | 0.033354 | -0.0143758 | 0.681373125 |
| rs1203943 | C | T | 1.83E-23 | 0.103531 | 0.010372 | 0.79178 | 287247 | 0.000346745 | 99.63519559 | 0.032516 | 0.00905992 | 0.789354338 |
| rs12091047 | T | C | 1.08E-25 | 0.0927867 | 0.00885449 | 0.316692 | 287247 | 0.00038214 | 109.8098582 | 0.026639 | -0.0765474 | 0.00972792 |
| rs12129508 | A | G | 4.49E-08 | 0.0690302 | 0.0126184 | 0.120108 | 287247 | 0.000104176 | 29.92723948 | 0.038426 | -0.0330554 | 0.424052863 |
| rs12206423 | T | C | 1.24E-08 | 0.0655998 | 0.0115195 | 0.14809 | 287247 | 0.000112884 | 32.429089 | 0.044507 | 0.0975803 | 0.020347919 |
| rs123378 | A | G | 3.85E-09 | -0.0502193 | 0.00852516 | 0.617779 | 287247 | 0.000120789 | 34.70029834 | 0.029463 | 0.00668228 | 0.82688307 |
| rs12506688 | T | C | 5.20E-14 | 0.0687936 | 0.00913992 | 0.281399 | 287247 | 0.000197183 | 56.65108878 | 0.030819 | 0.0253763 | 0.412555138 |
| rs12697352 | A | G | 1.49E-09 | -0.0514445 | 0.00850986 | 0.395739 | 287247 | 0.00012721 | 36.54517506 | 0.02992 | -0.00507586 | 0.869890925 |
| rs12736474 | A | G | 2.35E-09 | 0.053367 | 0.0089364 | 0.31216 | 287247 | 0.00012414 | 35.6629575 | 0.028754 | -0.0307283 | 0.31445029 |
| rs12756019 | A | G | 2.38E-11 | -0.0566832 | 0.00848487 | 0.60567 | 287247 | 0.000155344 | 44.6288104 | 0.028084 | 0.0358852 | 0.231209141 |
| rs13137589 | G | A | 4.42E-13 | -0.0627853 | 0.00866943 | 0.368031 | 287247 | 0.000182558 | 52.4483506 | 0.031244 | 0.0915778 | 0.001807008 |
| rs1317983 | C | T | 1.25E-27 | 0.0980504 | 0.00900134 | 0.680676 | 287247 | 0.000412904 | 118.6537267 | 0.032064 | -0.0287672 | 0.370841219 |
| rs13220783 | T | G | 3.99E-09 | 0.0499938 | 0.00849568 | 0.380325 | 287247 | 0.000120539 | 34.62844433 | 0.027706 | -0.0217386 | 0.455543653 |
| rs13447704 | C | T | 2.09E-08 | -0.14695 | 0.0262205 | 0.0268588 | 287247 | 0.000109334 | 31.40900791 | 0.096948 | 0.0404901 | 0.693676529 |
| rs1371867 | C | A | 5.71E-09 | 0.0487005 | 0.00836052 | 0.428582 | 287247 | 0.000118112 | 33.93104507 | 0.027824 | 0.00615601 | 0.828569037 |
| rs141686764 | G | A | 9.11E-09 | -0.182081 | 0.0316857 | 0.0187788 | 287247 | 0.000114947 | 33.02171493 | 0.093648 | 0.0149438 | 0.883236077 |
| rs1441172 | T | C | 3.43E-09 | -0.0533644 | 0.00903023 | 0.313428 | 287247 | 0.000121562 | 34.92228191 | 0.030665 | 0.0915988 | 0.001465008 |
| rs1534430 | T | C | 2.76E-11 | -0.056034 | 0.00841488 | 0.417775 | 287247 | 0.000154342 | 44.34088669 | 0.027522 | -0.0178046 | 0.536679492 |
| rs1788105 | G | A | 2.77E-08 | -0.0462256 | 0.00832056 | 0.547645 | 287247 | 0.000107438 | 30.86433873 | 0.025396 | 0.0733635 | 0.009021941 |
| rs2104047 | C | T | 1.20E-08 | 0.0514534 | 0.00902662 | 0.693491 | 287247 | 0.000113103 | 32.49187785 | 0.029838 | -0.00984339 | 0.746383448 |
| rs2111485 | G | A | 5.67E-18 | 0.0727979 | 0.00842671 | 0.583695 | 287247 | 0.000259749 | 74.63088343 | 0.030177 | -0.0735404 | 0.010784996 |
| rs2235544 | A | C | 4.66E-10 | -0.0519065 | 0.0083313 | 0.5448 | 287247 | 0.000135115 | 38.81637023 | 0.027896 | -0.0284825 | 0.306604836 |
| rs229528 | T | C | 6.96E-21 | 0.0790766 | 0.0084354 | 0.396388 | 287247 | 0.000305841 | 87.87828838 | 0.02647 | -0.0327154 | 0.244476996 |
| rs2402240 | A | G | 1.92E-18 | 0.0940805 | 0.0107375 | 0.176345 | 287247 | 0.000267191 | 76.76970224 | 0.035315 | 0.0304826 | 0.389971618 |
| rs244689 | G | A | 3.29E-14 | -0.0718721 | 0.00947387 | 0.749122 | 287247 | 0.00020032 | 57.55231573 | 0.040588 | -0.0740605 | 0.058578736 |
| rs2476601 | G | A | 1.71E-162 | -0.304028 | 0.0111921 | 0.854687 | 287247 | 0.002562325 | 737.9058911 | 0.042549 | 0.0618701 | 0.191775887 |
| rs2531989 | A | G | 5.90E-11 | -0.0695526 | 0.010625 | 0.817382 | 287247 | 0.000149159 | 42.85148111 | 0.036755 | -0.00494376 | 0.89633695 |
| rs2680296 | A | C | 1.17E-08 | 0.0473154 | 0.00829442 | 0.468365 | 287247 | 0.000113273 | 32.54093014 | 0.027221 | -0.0174422 | 0.540271427 |
| rs28391281 | C | T | 3.99E-09 | -0.0488629 | 0.00830364 | 0.546421 | 287247 | 0.000120535 | 34.627284 | 0.030557 | -0.0200905 | 0.532316588 |
| rs28582094 | G | A | 3.81E-09 | 0.0552873 | 0.00938328 | 0.261193 | 287247 | 0.000120846 | 34.71669602 | 0.032279 | 0.0164559 | 0.615725301 |
| rs2928167 | G | A | 3.52E-22 | -0.101973 | 0.0105297 | 0.199071 | 287247 | 0.000326393 | 93.78542973 | 0.04028 | 0.0039462 | 0.924739907 |
| rs2958154 | T | C | 7.35E-12 | -0.0616386 | 0.0089973 | 0.699391 | 287247 | 0.000163363 | 46.93297725 | 0.028282 | 0.0329055 | 0.274264754 |
| rs30233 | A | G | 4.58E-12 | -0.0582169 | 0.00841513 | 0.586872 | 287247 | 0.00016659 | 47.86008537 | 0.02885 | -0.0302353 | 0.293579685 |
| rs35717611 | T | C | 1.26E-08 | 0.0487793 | 0.0085697 | 0.378643 | 287247 | 0.000112781 | 32.39939242 | 0.032296 | 0.00107043 | 0.974372908 |
| rs3946137 | G | A | 1.22E-09 | 0.0526375 | 0.00866007 | 0.349188 | 287247 | 0.000128599 | 36.94406503 | 0.030052 | -0.0169671 | 0.590391369 |
| rs41177 | A | G | 2.48E-09 | -0.0504861 | 0.00846668 | 0.404374 | 287247 | 0.000123768 | 35.55611735 | 0.02978 | 0.055605 | 0.055005992 |
| rs4409785 | C | T | 9.15E-16 | 0.0882346 | 0.0109776 | 0.165925 | 287247 | 0.000224859 | 64.60409017 | 0.035693 | -0.0450643 | 0.245135946 |
| rs4575545 | A | G | 1.12E-20 | -0.0852985 | 0.00914861 | 0.302816 | 287247 | 0.000302542 | 86.92997119 | 0.029407 | -0.0184227 | 0.55065806 |
| rs4606850 | T | C | 2.55E-08 | 0.0616895 | 0.0110754 | 0.165369 | 287247 | 0.000107994 | 31.0242032 | 0.034917 | -0.00911441 | 0.80288196 |
| rs4704397 | A | G | 8.89E-56 | 0.131555 | 0.00836138 | 0.41002 | 287247 | 0.000861052 | 247.5459192 | 0.028666 | 0.015128 | 0.602523514 |
| rs4804433 | T | G | 2.49E-17 | -0.0754197 | 0.00890613 | 0.692424 | 287247 | 0.00024959 | 71.71145216 | 0.032477 | 0.0177099 | 0.604354834 |
| rs4853459 | C | T | 8.01E-43 | -0.132855 | 0.0096853 | 0.769419 | 287247 | 0.000654621 | 188.1597073 | 0.031525 | 0.0277032 | 0.408059037 |
| rs56983610 | A | C | 5.30E-18 | -0.108696 | 0.0125708 | 0.13032 | 287247 | 0.000260215 | 74.76498787 | 0.035573 | -0.0245058 | 0.517075547 |
| rs57652885 | T | C | 2.65E-09 | -0.112845 | 0.0189593 | 0.0530945 | 287247 | 0.000123314 | 35.42558418 | 0.114987 | 0.143566 | 0.195124139 |
| rs61759532 | T | C | 1.20E-12 | 0.0747524 | 0.0105202 | 0.188962 | 287247 | 0.00017574 | 50.48929016 | 0.038305 | -0.0189005 | 0.641658571 |
| rs61916675 | G | A | 3.01E-11 | 0.0590178 | 0.00888003 | 0.316646 | 287247 | 0.00015375 | 44.17068586 | 0.030486 | 0.0331852 | 0.274631912 |
| rs6724363 | T | G | 7.67E-24 | 0.0864868 | 0.00859042 | 0.624805 | 287247 | 0.000352746 | 101.3602885 | 0.030484 | -0.00253877 | 0.935444013 |
| rs6831973 | C | T | 4.47E-12 | 0.0577679 | 0.00834607 | 0.555424 | 287247 | 0.000166756 | 47.90778488 | 0.027617 | -0.00484325 | 0.863861064 |
| rs6914622 | T | G | 4.25E-14 | 0.0716868 | 0.00949102 | 0.254521 | 287247 | 0.000198569 | 57.04920385 | 0.02923 | -0.0262341 | 0.396264348 |
| rs7043516 | C | A | 1.45E-18 | -0.0998544 | 0.0113558 | 0.166059 | 287247 | 0.000269108 | 77.32072524 | 0.038431 | -0.0299109 | 0.468064819 |
| rs705702 | G | A | 1.95E-18 | 0.0785362 | 0.00896516 | 0.302645 | 287247 | 0.000267087 | 76.73979851 | 0.028288 | -0.0570191 | 0.064687445 |
| rs713427 | C | T | 8.38E-09 | 0.0571529 | 0.00992134 | 0.221764 | 287247 | 0.000115513 | 33.18431475 | 0.037748 | 0.0217096 | 0.571282604 |
| rs71641308 | T | C | 1.75E-09 | 0.0837373 | 0.0139108 | 0.0964292 | 287247 | 0.000126131 | 36.23520059 | 0.074618 | 0.202566 | 0.001807008 |
| rs72796365 | T | C | 4.49E-08 | 0.138961 | 0.0254015 | 0.0260409 | 287247 | 0.000104176 | 29.92706392 | 0.104056 | 0.0698133 | 0.516606659 |
| rs72866766 | C | T | 5.71E-09 | 0.0810729 | 0.0139181 | 0.0946304 | 287247 | 0.000118109 | 33.93036155 | 0.049475 | -0.0118793 | 0.821630022 |
| rs7321973 | A | C | 1.73E-19 | 0.0814066 | 0.00901603 | 0.295517 | 287247 | 0.000283733 | 81.52400613 | 0.034044 | 0.0233375 | 0.497440338 |
| rs76169968 | A | G | 2.85E-11 | -0.0943365 | 0.0141771 | 0.0989575 | 287247 | 0.000154121 | 44.27735703 | 0.048038 | -0.0333676 | 0.523136473 |
| rs76653830 | T | C | 2.20E-08 | 0.0754617 | 0.0134855 | 0.102668 | 287247 | 0.000108997 | 31.31237712 | 0.074172 | 0.137421 | 0.046834943 |
| rs774121 | C | T | 1.35E-12 | -0.0673027 | 0.00949342 | 0.749377 | 287247 | 0.00017494 | 50.25931449 | 0.0309 | 0.0234488 | 0.472743972 |
| rs7844425 | G | T | 2.15E-09 | -0.0520926 | 0.00870196 | 0.355567 | 287247 | 0.000124741 | 35.8356164 | 0.029687 | 0.0176965 | 0.555726363 |
| rs7902146 | T | C | 3.71E-16 | -0.0774861 | 0.00951006 | 0.750339 | 287247 | 0.00023106 | 66.38623938 | 0.028764 | 0.0835436 | 0.009646061 |
| rs794999 | G | A | 1.02E-11 | -0.0666523 | 0.00979602 | 0.77136 | 287247 | 0.000161141 | 46.2943436 | 0.031208 | 0.0293475 | 0.376637016 |
| rs8006310 | G | A | 9.68E-11 | -0.0536927 | 0.00829636 | 0.504402 | 287247 | 0.000145793 | 41.88439873 | 0.027635 | -0.00406826 | 0.886578918 |
| rs8087237 | A | C | 6.54E-09 | -0.0482406 | 0.00831393 | 0.490285 | 287247 | 0.000117194 | 33.66740555 | 0.028305 | 0.0335856 | 0.232511266 |
| rs8193 | T | C | 4.41E-11 | 0.0570366 | 0.00865554 | 0.352657 | 287247 | 0.000151146 | 43.42260746 | 0.029121 | 0.0381172 | 0.186116124 |
| rs897586 | A | G | 1.87E-12 | -0.0630711 | 0.00895376 | 0.318214 | 287247 | 0.000172711 | 49.61887217 | 0.030378 | 0.0454035 | 0.128725919 |
| rs9292 | G | A | 1.18E-09 | -0.140954 | 0.0231718 | 0.968488 | 287247 | 0.000128802 | 37.00260018 | 0.091713 | -0.0414452 | 0.667850107 |
| rs9975418 | G | A | 1.06E-08 | 0.0613504 | 0.010725 | 0.179825 | 287247 | 0.000113903 | 32.72179894 | 0.031953 | -0.0194836 | 0.562825803 |

Abbreviations: A1, effect allele; A2, other allele; beta, effect size; se, standard error of beta; PVE, the proportion of variance explained by individual SNP. There are totally 85 valid instrumental SNP variables for hypothyroidism, with the PVE being 1.94%.The overall F statistic for the 85 SNPs is 66.80 (PVE=1.94%, k=85 and N=287247).

**Supplementary Table S12.** The instrumental SNP variables for lung adenocarcinoma on hypothyroidism

| rsid | A1 | A2 | lung adenocarcinoma | | | | | | | hypothyroidism | | |
| --- | --- | --- | --- | --- | --- | --- | --- | --- | --- | --- | --- | --- |
|  |  |  | P.x | beta.x | se.x | eaf | N | PVE | F | P.y | se.y | beta.y |
| rs11813268 | T | C | 2.45E-06 | 0.178117 | 0.04351 | 0.175737 | 18336 | 0.000913127 | 16.75657303 | 0.836229 | 0.01206 | 0.00249305 |
| rs138019214 | C | T | 2.57E-06 | 0.924129 | 0.410734 | 0.00930357 | 18336 | 0.000276007 | 5.061701328 | 0.541198 | 0.074809 | 0.0457086 |
| rs144483781 | G | A | 2.99E-06 | 0.446618 | 0.136178 | 0.0287626 | 18336 | 0.000586272 | 10.75501867 | 0.0383911 | 0.0643128 | 0.13317 |
| rs2736100 | A | C | 5.13E-15 | -0.219362 | 0.021892 | 0.485585 | 18336 | 0.005445976 | 100.3932565 | 0.054325 | 0.00828549 | 0.0159433 |
| rs300219 | G | T | 6.97E-07 | -0.748504 | 0.275871 | 0.987397 | 18336 | 0.000401326 | 7.360863759 | 0.837943 | 0.0549902 | 0.0112469 |
| rs35271424 | A | G | 1.08E-06 | 0.557847 | 0.1789 | 0.0149378 | 18336 | 0.000529999 | 9.722146238 | 0.0526514 | 0.0369913 | 0.0716807 |
| rs35953480 | A | G | 2.28E-06 | 0.403263 | 0.117509 | 0.0376051 | 18336 | 0.000641876 | 11.77571047 | 0.716925 | 0.0332701 | 0.0120628 |
| rs446975 | T | G | 4.67E-06 | -0.226023 | 0.037506 | 0.119269 | 18336 | 0.001976696 | 36.31252077 | 0.862944 | 0.0122485 | -0.00211443 |
| rs4592420 | C | T | 2.04E-07 | 0.170415 | 0.026772 | 0.751276 | 18336 | 0.002204909 | 40.51414069 | 0.187449 | 0.00932888 | 0.012297 |
| rs55731496 | C | A | 6.24E-08 | -0.316652 | 0.040249 | 0.0885607 | 18336 | 0.003364234 | 61.88806558 | 0.54319 | 0.015939 | -0.00969082 |
| rs56077333 | A | C | 2.91E-24 | 0.308928 | 0.040166 | 0.344802 | 18336 | 0.003215836 | 59.14935382 | 0.819332 | 0.0088167 | -0.00201377 |
| rs73069440 | C | T | 3.46E-06 | 0.267887 | 0.071287 | 0.0905647 | 18336 | 0.000769562 | 14.12001725 | 0.960957 | 0.0126937 | -0.000621391 |
| rs77151940 | A | C | 2.27E-06 | 0.723977 | 0.272712 | 0.0129059 | 18336 | 0.000384211 | 7.046827956 | 0.915468 | 0.0483989 | -0.00513725 |
| rs79530271 | A | G | 2.92E-06 | 0.775671 | 0.307411 | 0.0134101 | 18336 | 0.000347105 | 6.366033636 | 0.330091 | 0.0240448 | 0.0234179 |

Abbreviations: A1, effect allele; A2, other allele; beta, effect size; se, standard error of beta; PVE, the proportion of variance explained by individual SNP. There are totally 14 valid instrumental SNP variables for lung adenocarcinoma, with the PVE being 2.11%.The overall F statistic for the 14 SNPs is 28.15 (PVE=2.11%, k=14 and N=18336).

**Supplementary Table S13.** The instrumental SNP variables for squamous cell lung cancer on hypothyroidism

| rsid | A1 | A2 | squamous cell lung cancer | | | | | | | hypothyroidism | | |
| --- | --- | --- | --- | --- | --- | --- | --- | --- | --- | --- | --- | --- |
|  |  |  | P.x | beta.x | se.x | eaf | N | PVE | F | P.y | se.y | beta.y |
| rs115557325 | A | G | 3.49E-06 | -0.309669 | 0.045874 | 0.0619701 | 18313 | 0.002482124 | 45.56326213 | 0.429849 | 0.026238 | 0.0207136 |
| rs11571818 | C | T | 6.28E-09 | 0.866457 | 0.307405 | 0.0112708 | 18313 | 0.000433635 | 7.9437349 | 0.216883 | 0.0430437 | -0.0531529 |
| rs117745457 | A | G | 4.09E-06 | 0.667868 | 0.245942 | 0.00990189 | 18313 | 0.000402514 | 7.373410908 | 0.330734 | 0.0292254 | -0.0284257 |
| rs12914385 | T | C | 2.30E-25 | 0.299604 | 0.037745 | 0.40799 | 18313 | 0.003428667 | 62.99831337 | 0.816063 | 0.00863778 | -0.00200924 |
| rs1333040 | T | C | 3.95E-06 | -0.13073 | 0.03139 | 0.567469 | 18313 | 0.000946231 | 17.34284961 | 0.786383 | 0.00830047 | 0.00224952 |
| rs142411553 | T | C | 1.22E-06 | 0.72532 | 0.26742 | 0.014365 | 18313 | 0.000401548 | 7.355701621 | 0.11471 | 0.0311007 | 0.0490574 |
| rs27996 | G | A | 1.84E-08 | -0.165813 | 0.024244 | 0.419351 | 18313 | 0.002547776 | 46.77148622 | 0.903657 | 0.00833238 | -0.00100858 |
| rs28987111 | A | G | 2.80E-06 | 0.513408 | 0.164676 | 0.0208139 | 18313 | 0.000530487 | 9.718902196 | 0.0881576 | 0.0235288 | -0.0401212 |
| rs4079367 | T | C | 1.39E-06 | 0.163267 | 0.038512 | 0.476117 | 18313 | 0.000980436 | 17.97037942 | 0.00885483 | 0.00841657 | 0.0220313 |
| rs446975 | T | G | 9.56E-07 | -0.223554 | 0.034873 | 0.121322 | 18313 | 0.002238996 | 41.09025512 | 0.862944 | 0.0122485 | -0.00211443 |
| rs55791720 | C | T | 1.05E-07 | 0.150858 | 0.023715 | 0.520672 | 18313 | 0.002204816 | 40.46159519 | 0.954308 | 0.008307 | 0.000475976 |
| rs76967445 | A | C | 1.69E-06 | -0.14995 | 0.02613 | 0.281009 | 18313 | 0.001795041 | 32.92810121 | 0.104589 | 0.00893228 | -0.0144971 |

Abbreviations: A1, effect allele; A2 other allele; beta, effect size; se, standard error of beta; PVE, the proportion of variance explained by individual SNP. There are totally 12 valid instrumental SNP variables for squamous cell lung cancer, with the PVE being 1.84%.The overall F statistic for the 12 SNPs is 28.57 (PVE=1.84%, k=12 and N=18313).

## 1.2Supplementary **Figures**

**Supplementary Figure S1.** Forest plot, leave-one-out plot, funnel plot and scatter plot for the MR analysis inferring the causal effect of hypothyroidism on lung cancer

**
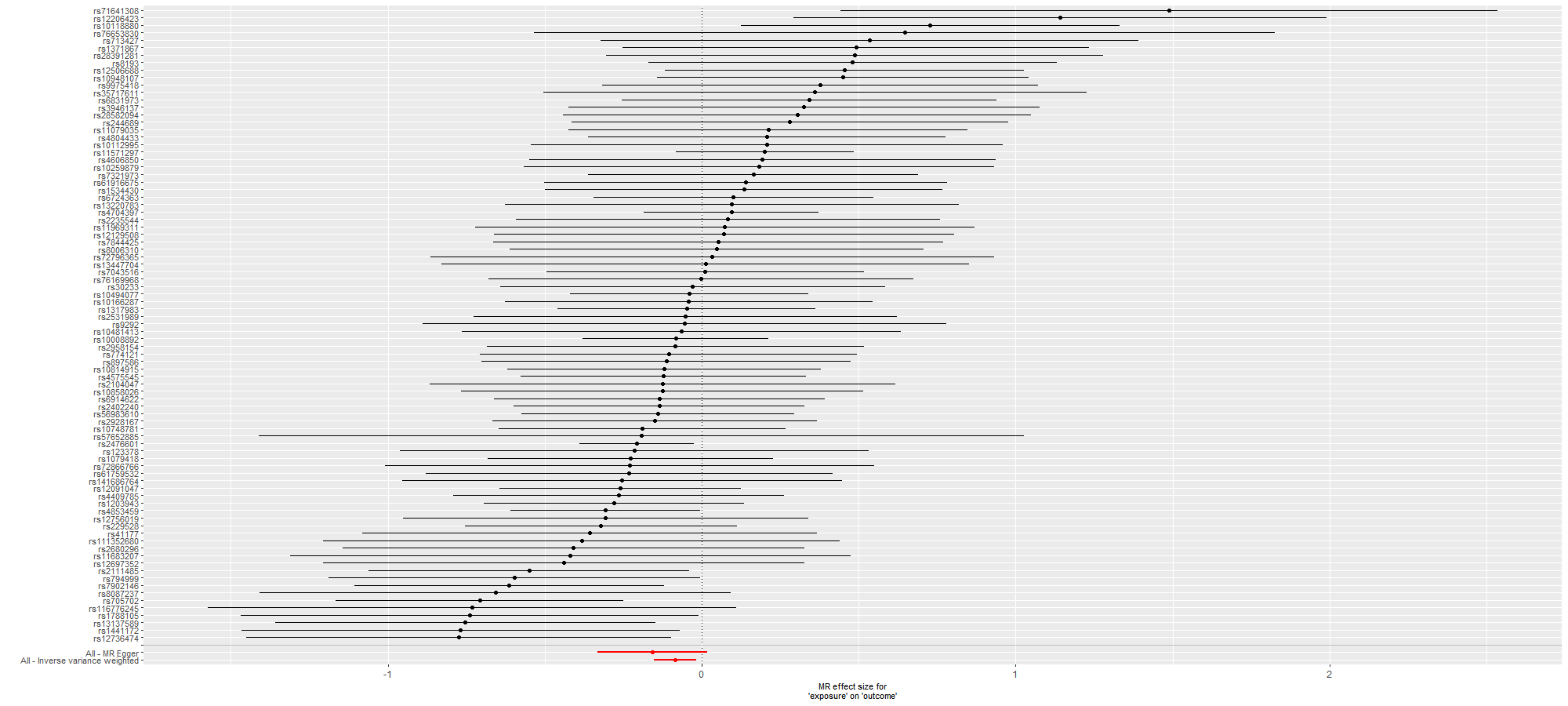

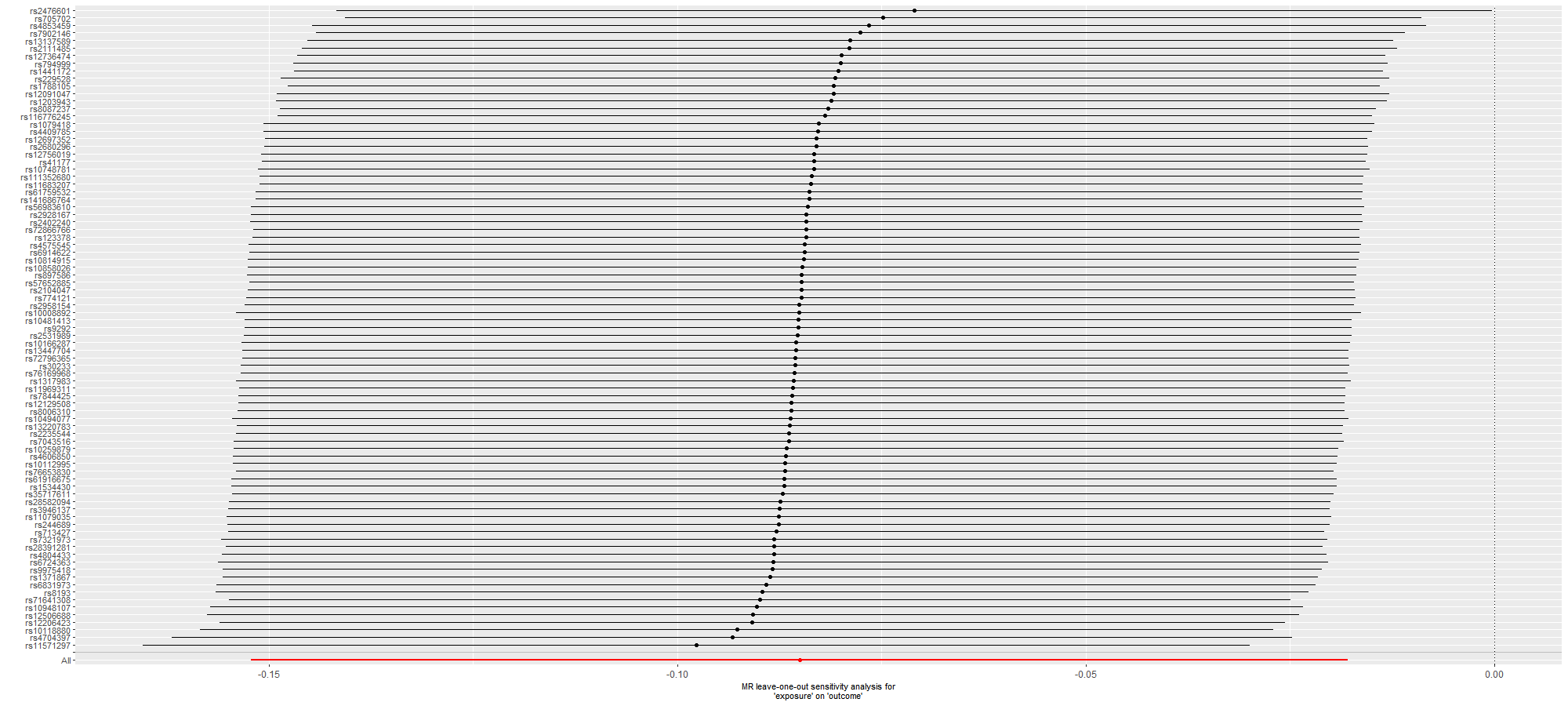

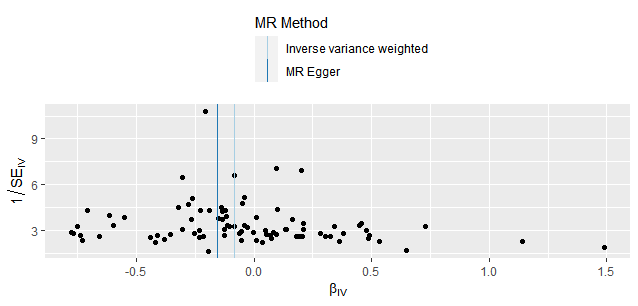

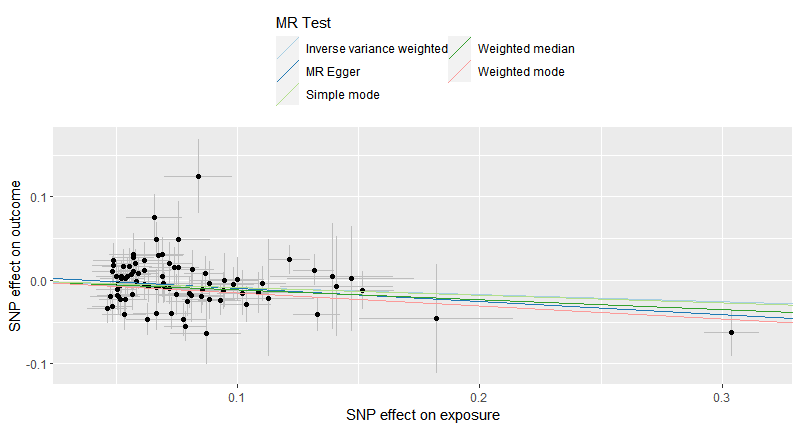
**

**Supplementary Figure S2.** Forest plot, leave-one-out plot, funnel plot and scatter plot for the MR analysis inferring the causal effect of hyperthyroidism on lung cancer

**
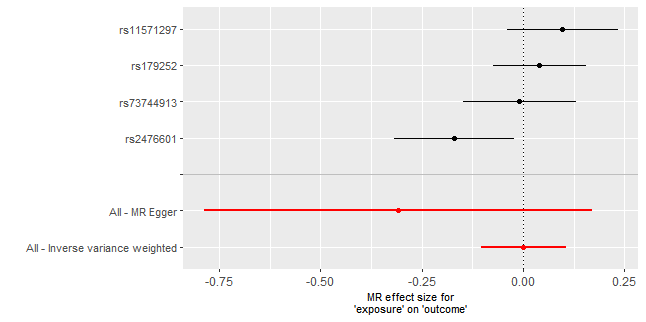

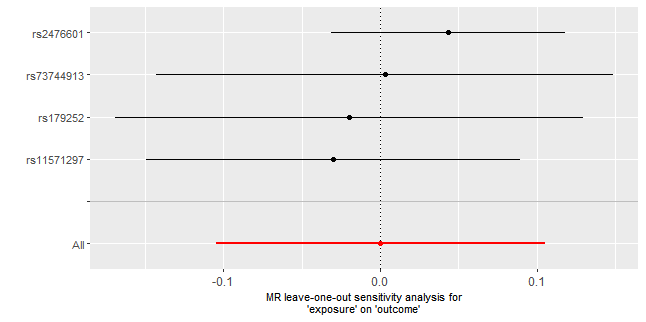

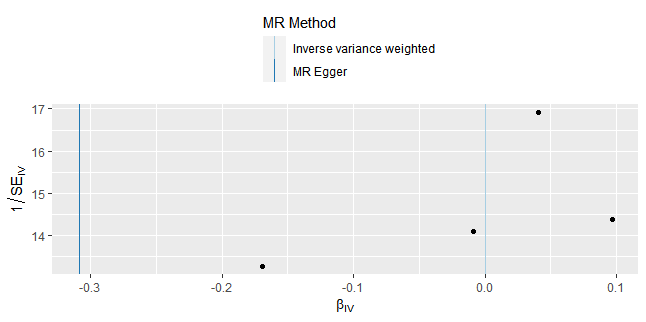

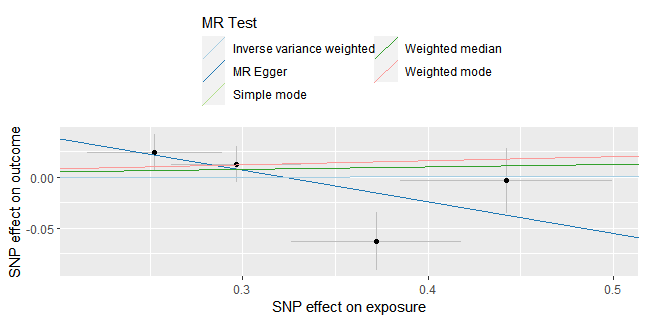
**

**Supplementary Figure S3.** Forest plot, leave-one-out plot, funnel plot and scatter plot for the MR analysis inferring the causal effect of TSH on lung cancer


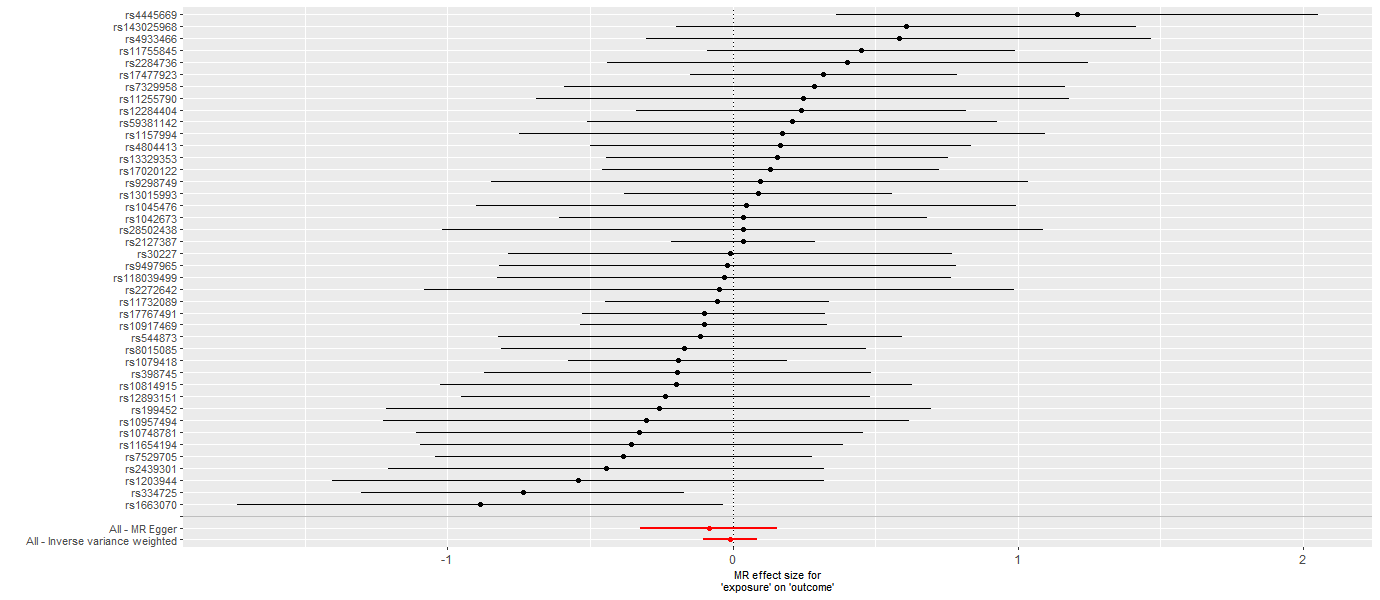

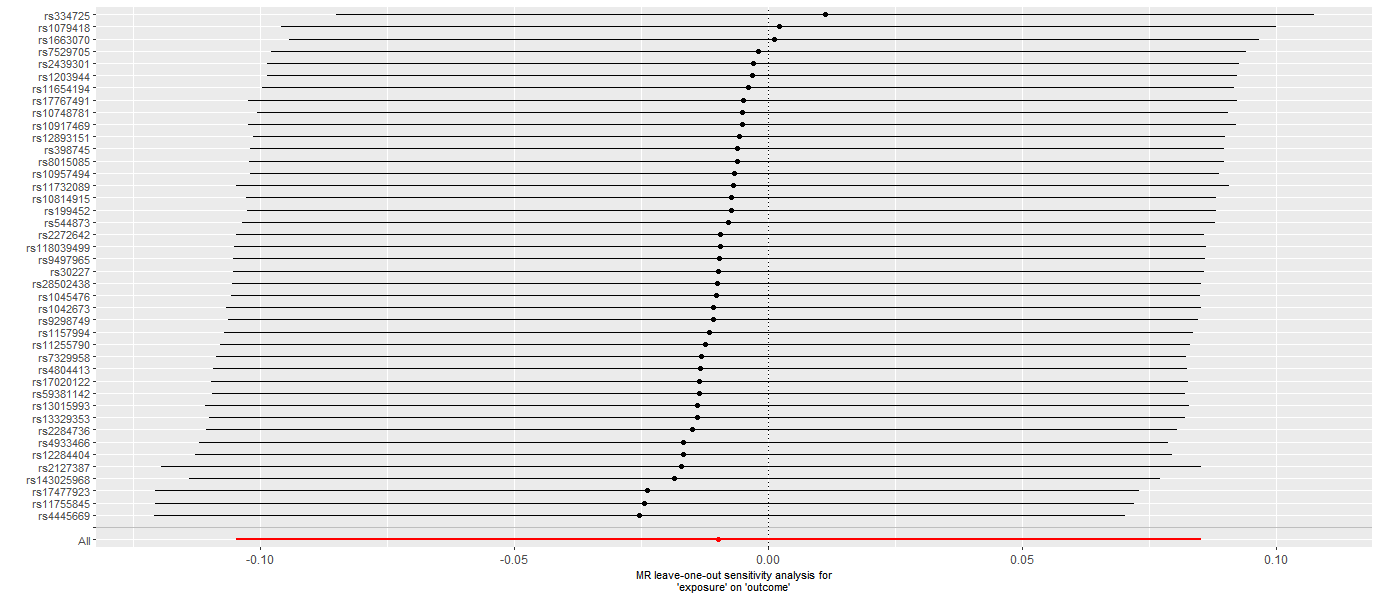

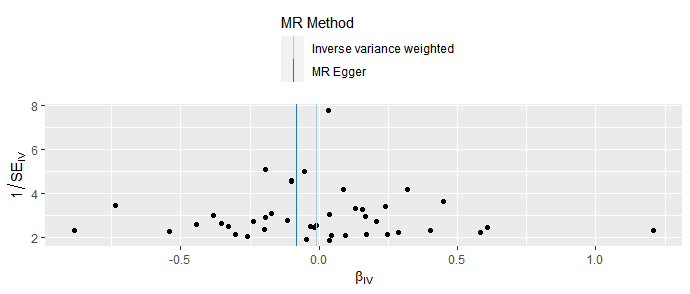

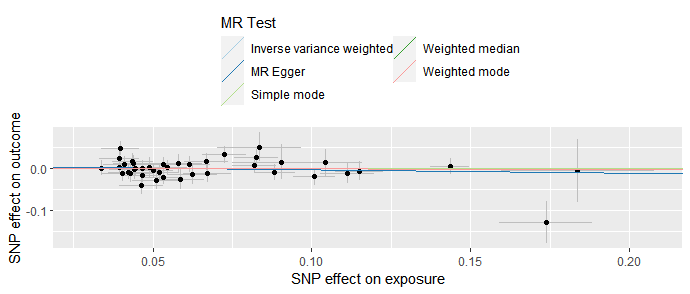


**Supplementary Figure S4.** Forest plot, leave-one-out plot, funnel plot and scatter plot for the MR analysis inferring the causal effect of FT4 on lung cancer


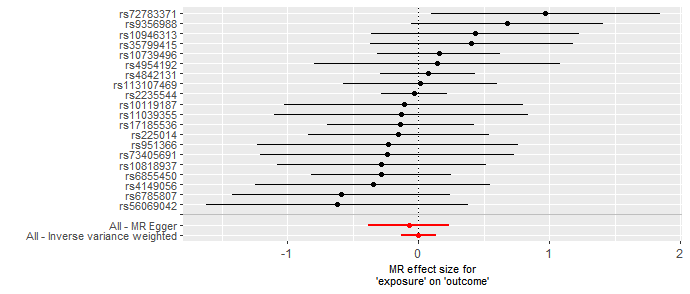

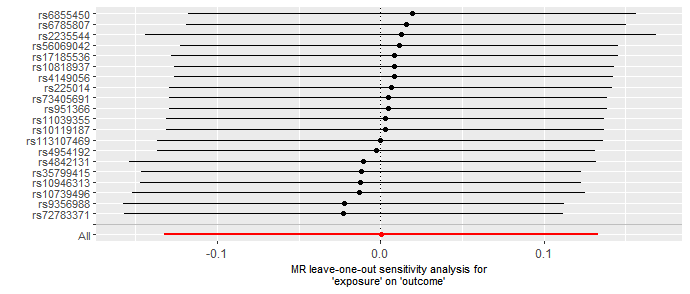

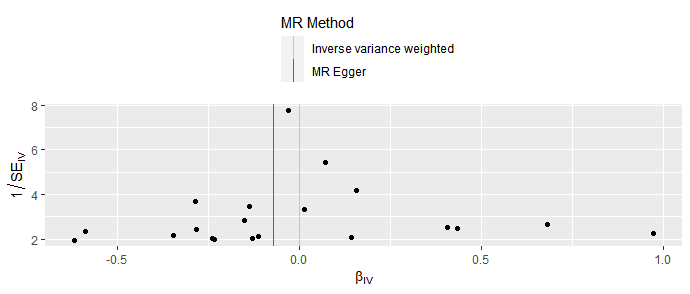

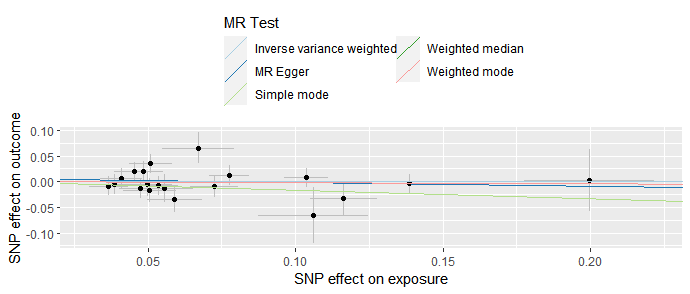


**Supplementary Figure S5.** Forest plot, leave-one-out plot, funnel plot and scatter plot for the MR analysis inferring the causal effect of lung cancer on hypothyroidism


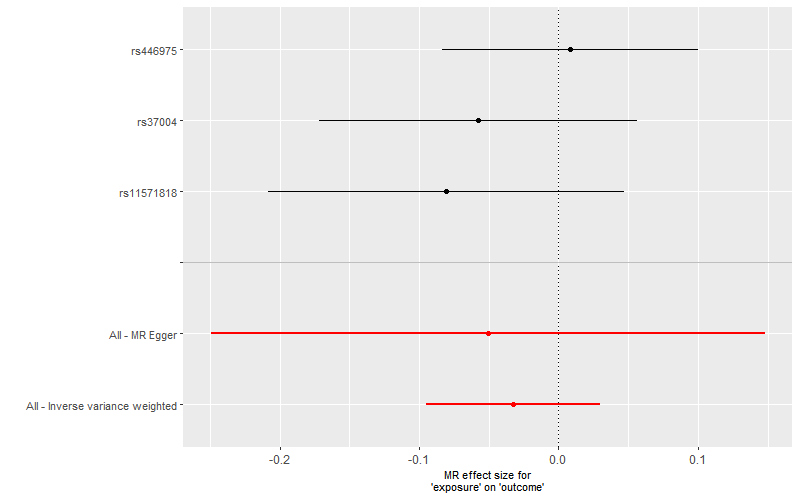

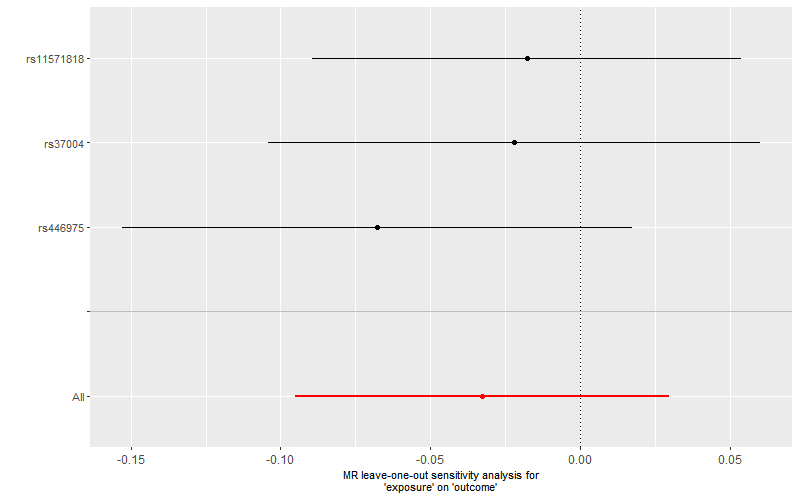

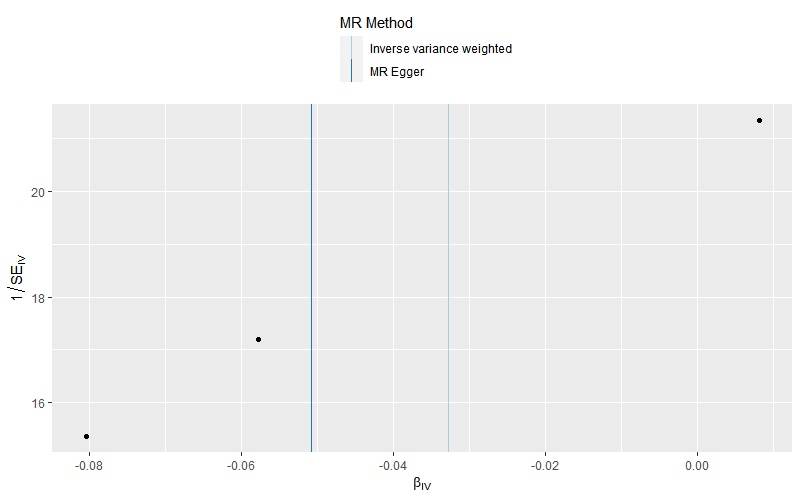

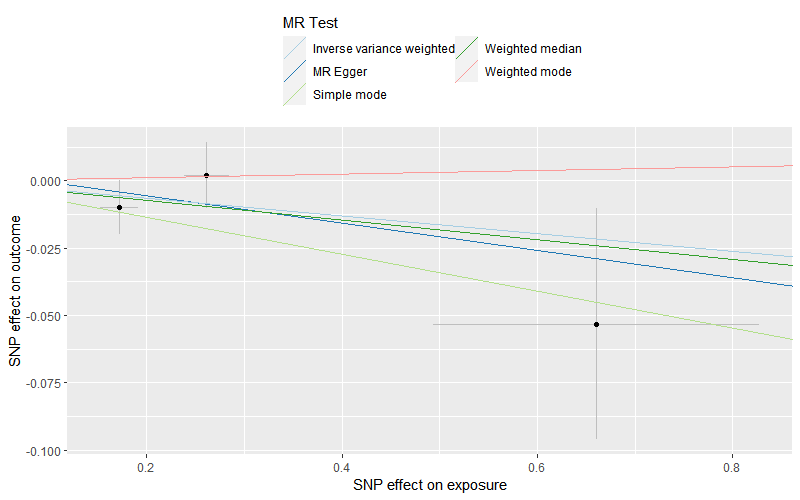


**Supplementary Figure S6.** Forest plot, leave-one-out plot, funnel plot and scatter plot for the MR analysis inferring the causal effect of lung cancer on hyperthyroidism


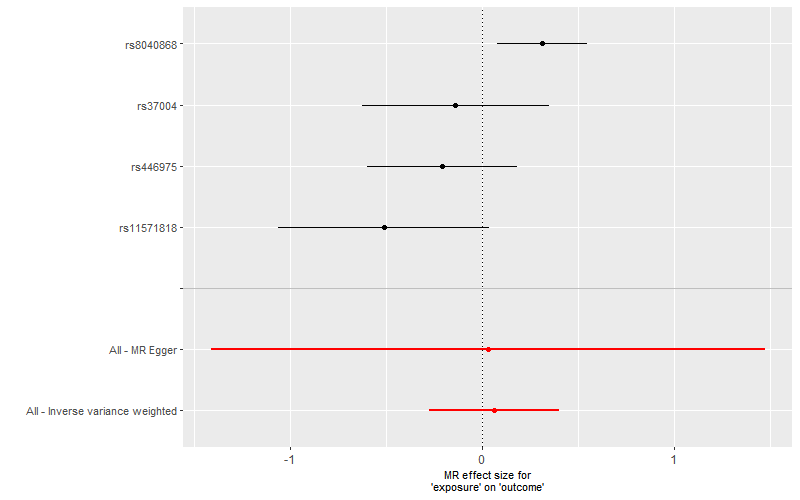

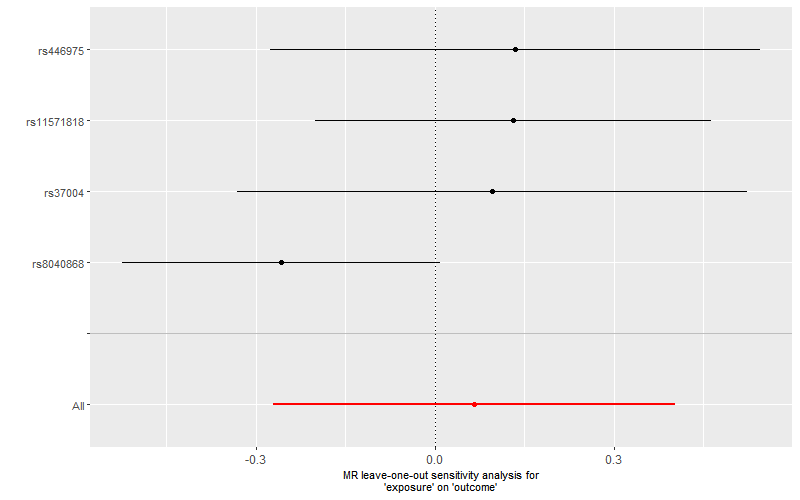

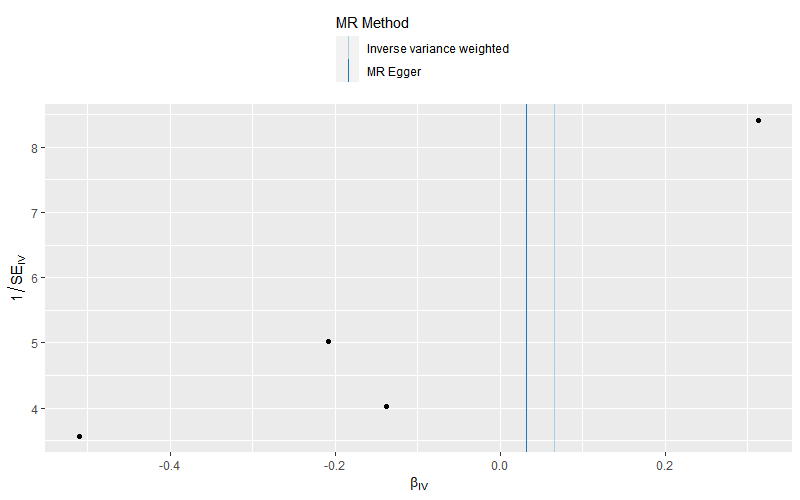

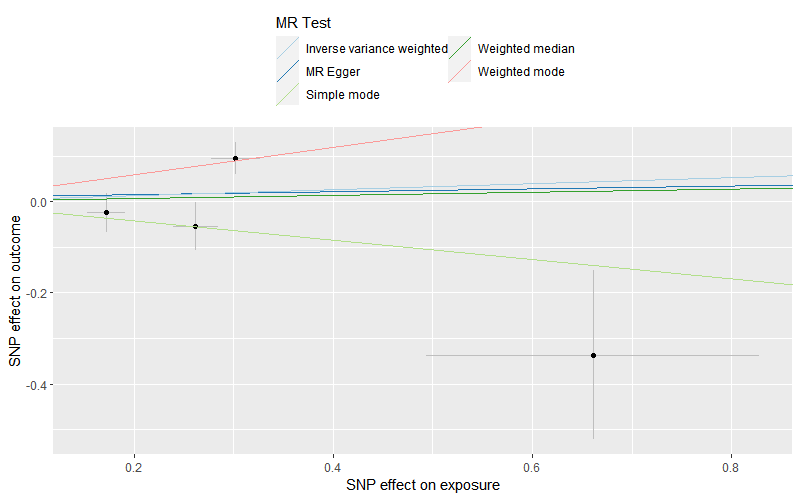


**Supplementary Figure S7.** Forest plot, leave-one-out plot, funnel plot and scatter plot for the MR analysis inferring the causal effect of lung cancer on TSH


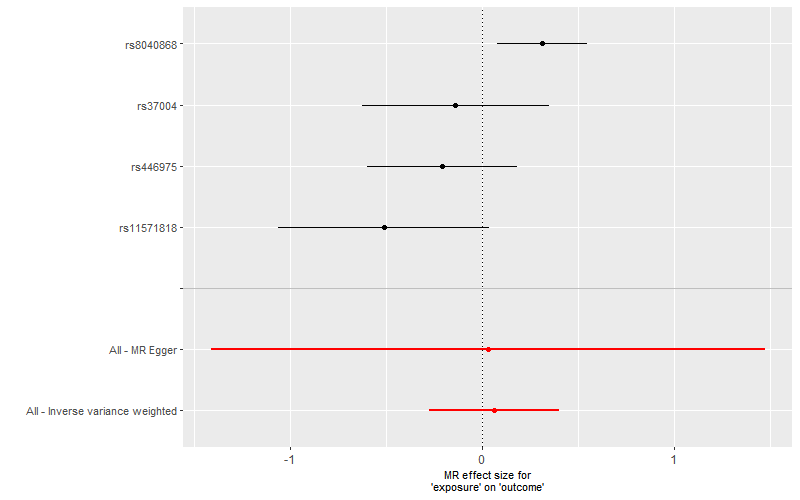

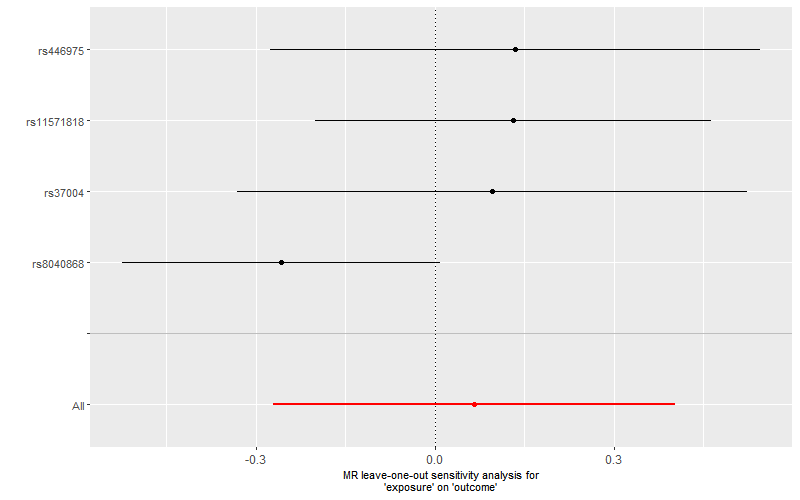

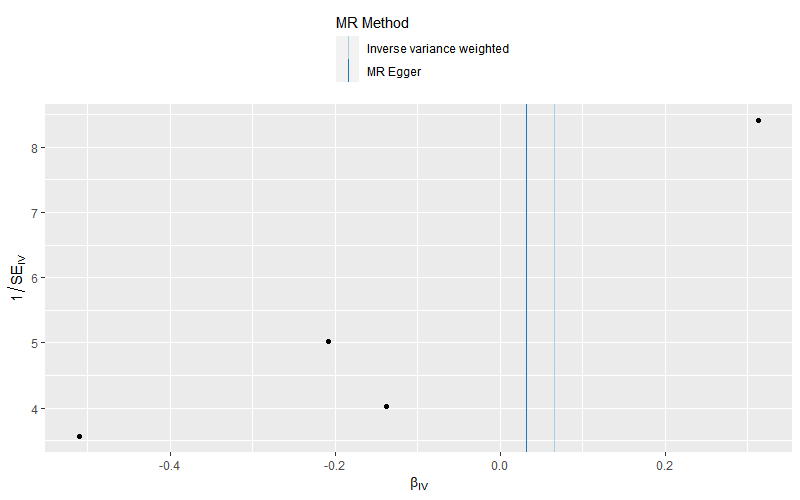

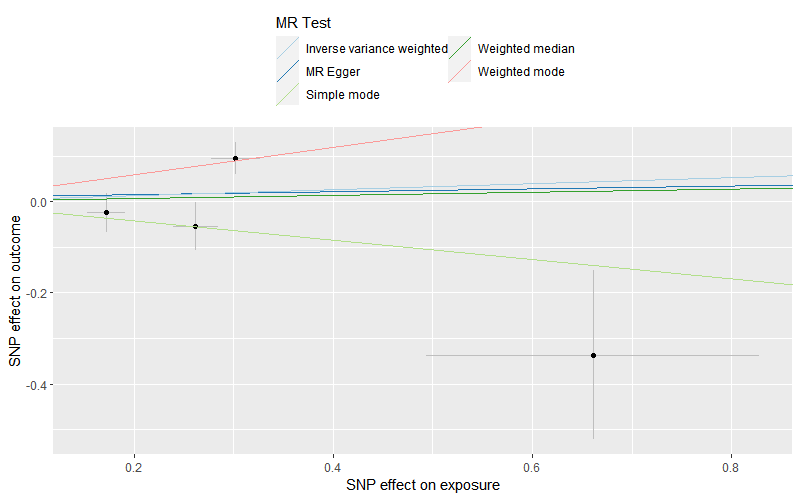


**Supplementary Figure S8.** Forest plot, leave-one-out plot, funnel plot and scatter plot for the MR analysis inferring the causal effect of lung cancer on FT4


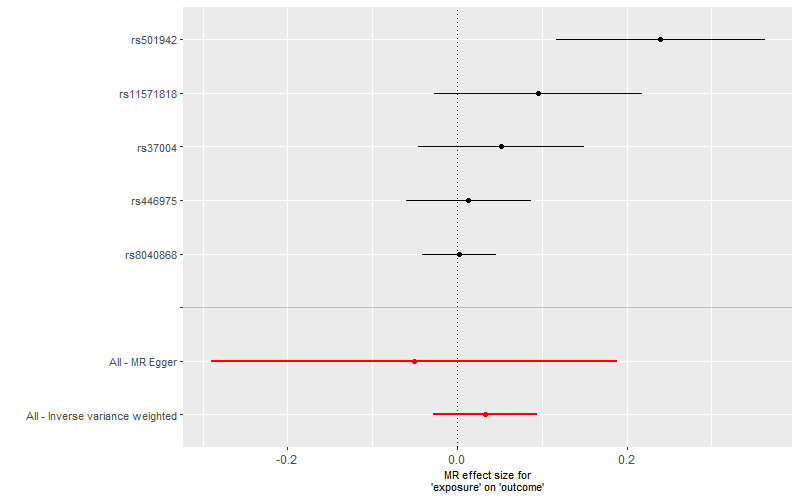

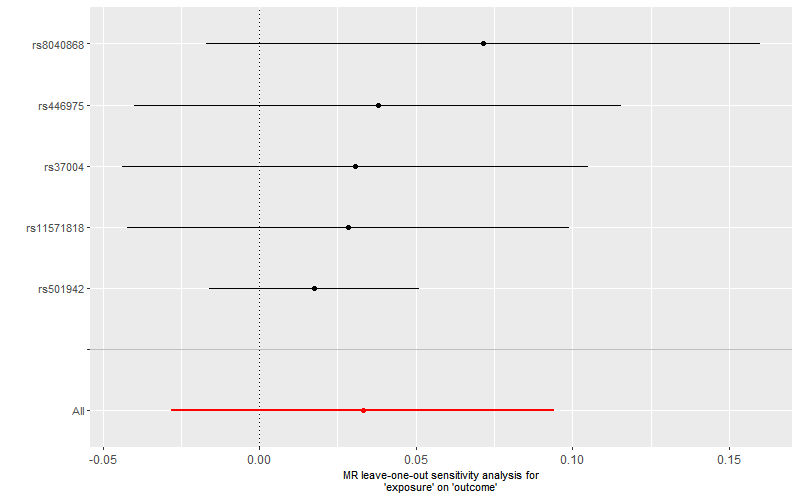

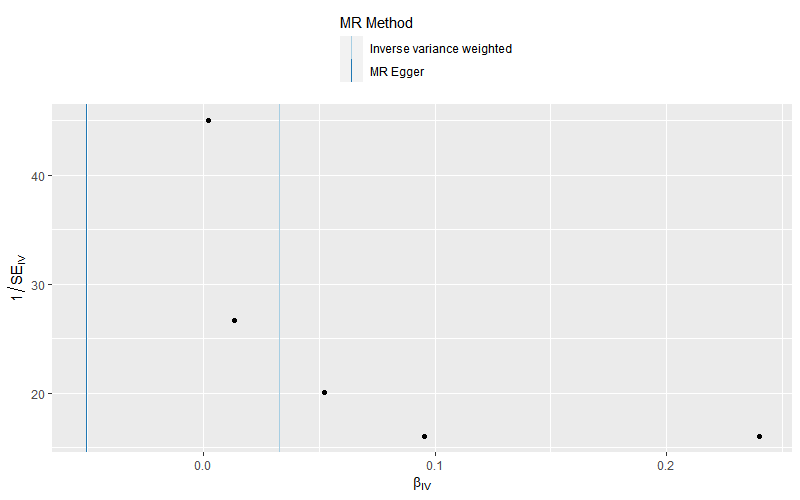

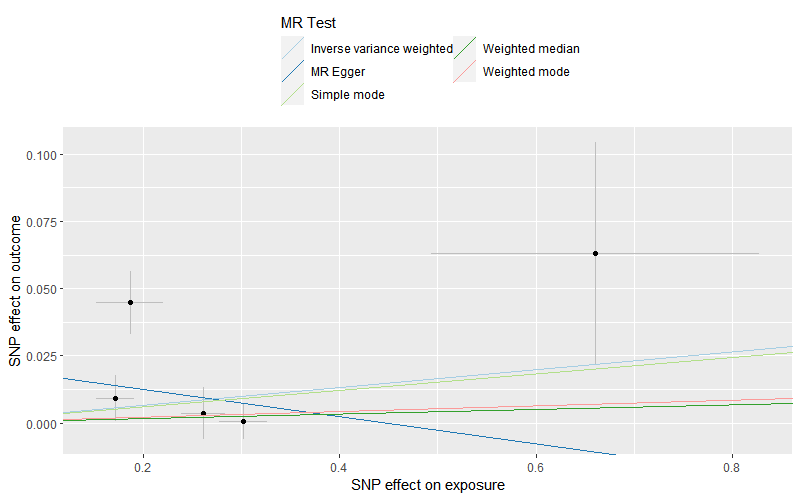


**Supplementary Figure S9.** Forest plot, leave-one-out plot, funnel plot and scatter plot for the MR analysis inferring the causal effect of hypothyroidism on lung adenocarcinoma


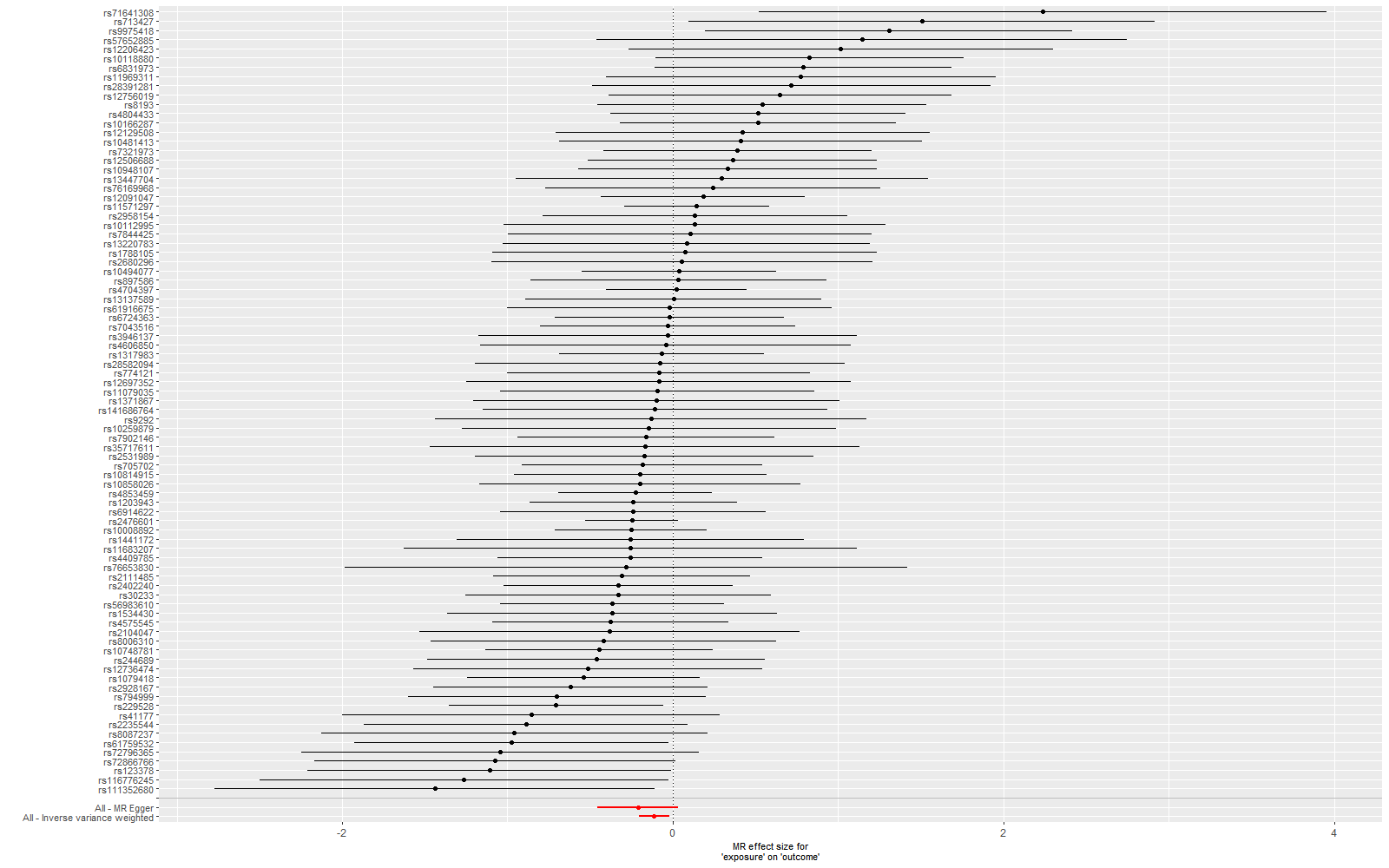

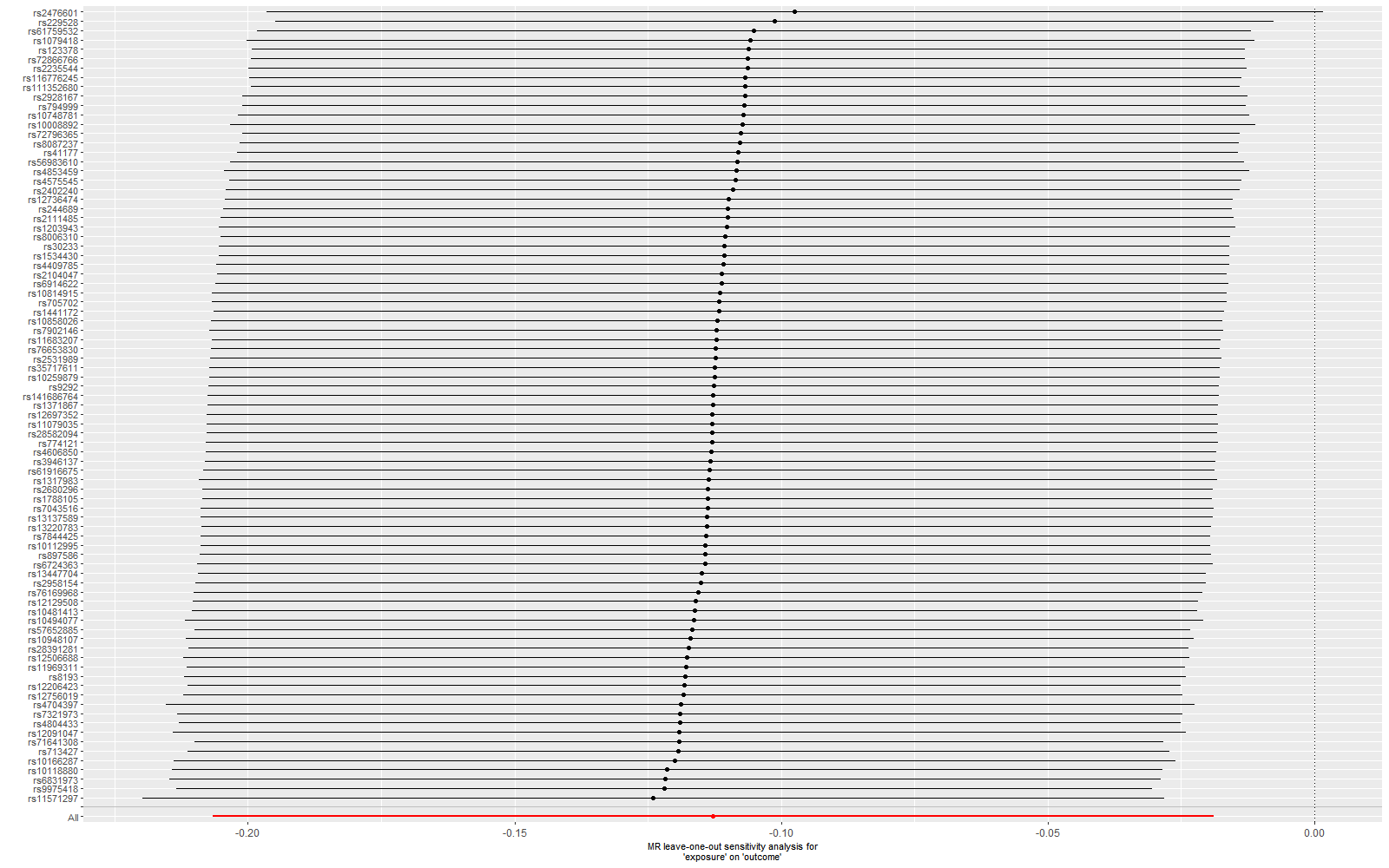

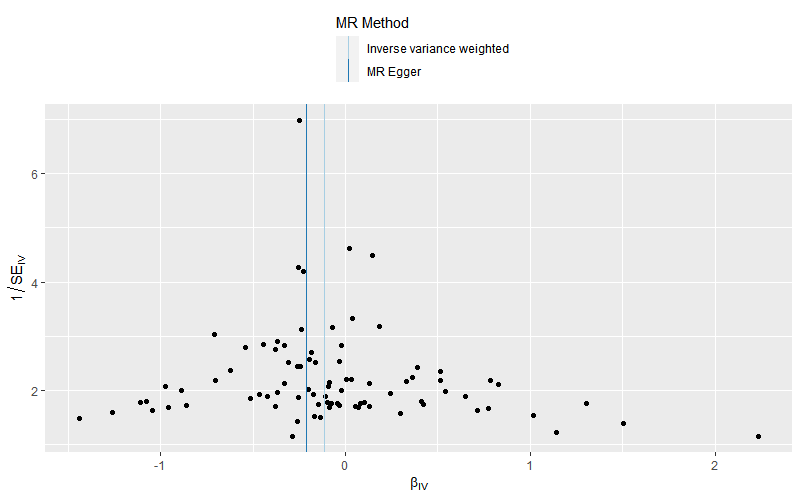

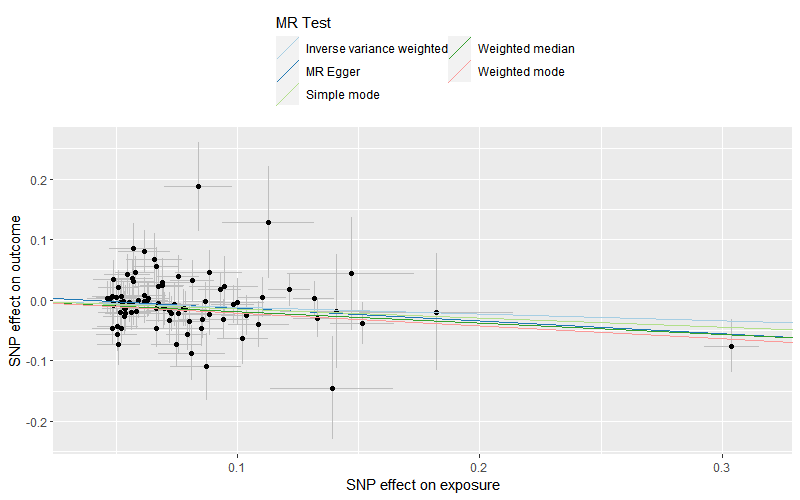


**Supplementary Figure S10.** Forest plot, leave-one-out plot, funnel plot and scatter plot for the MR analysis inferring the causal effect of hypothyroidism on Squamous cell lung cancer


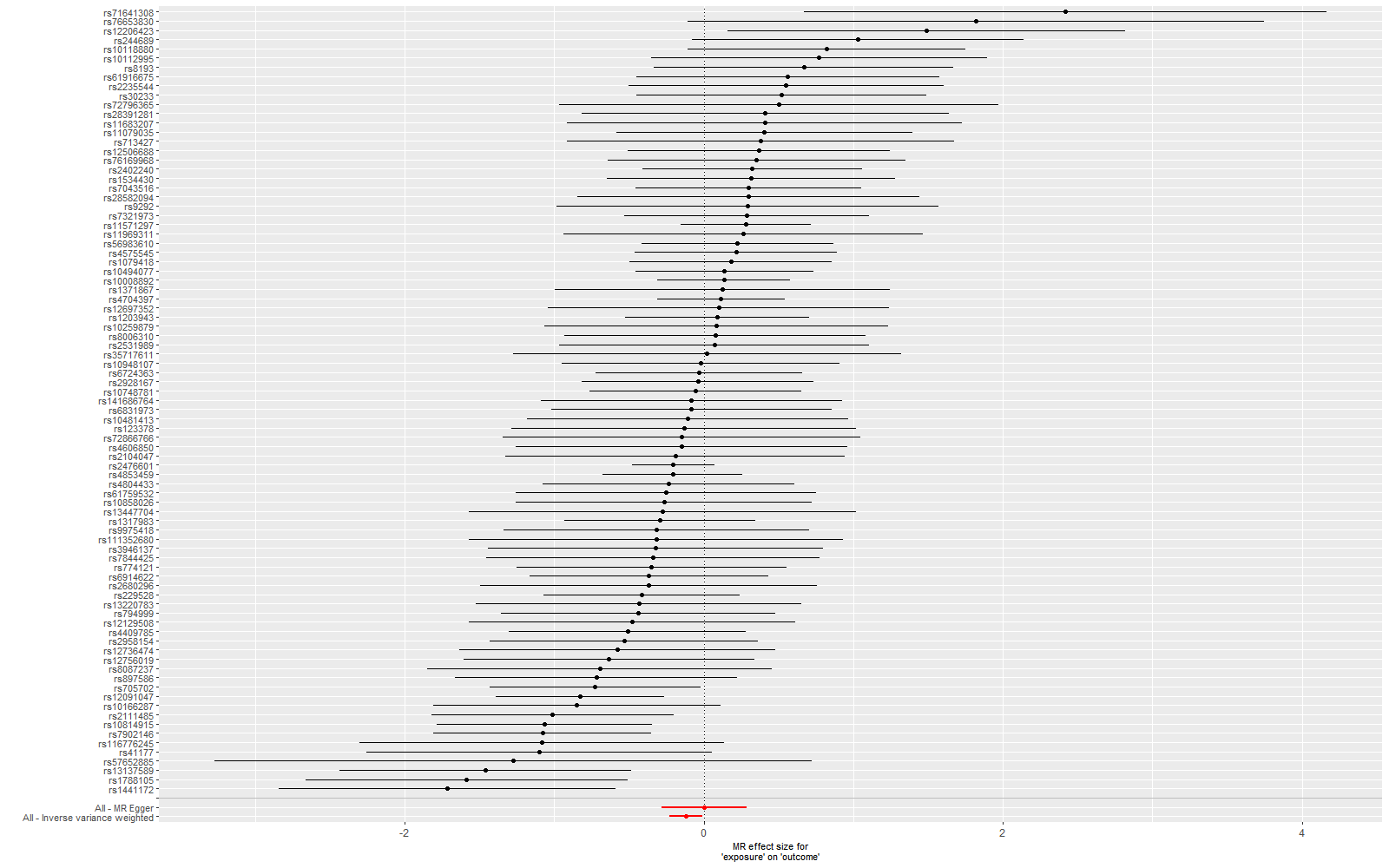

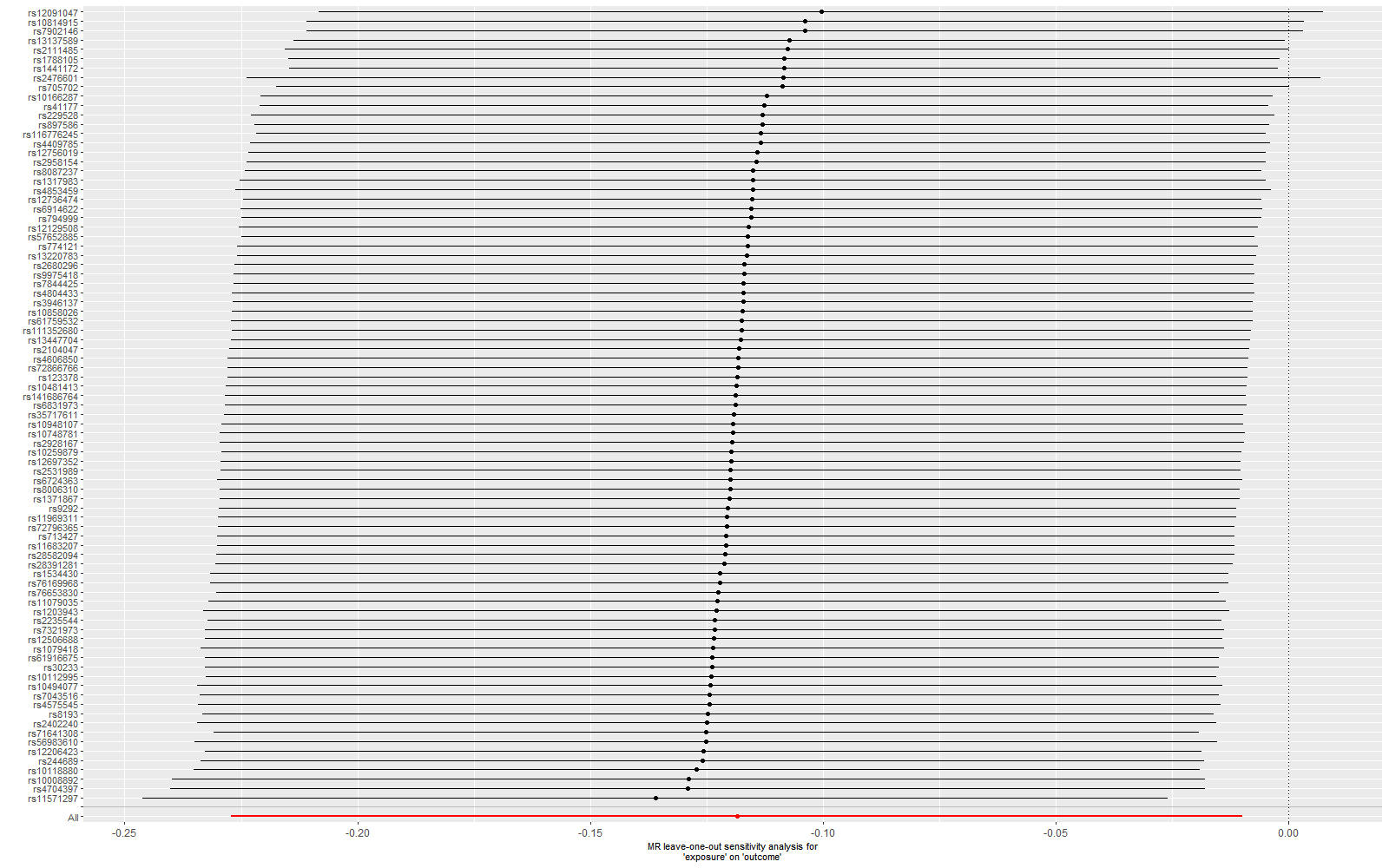

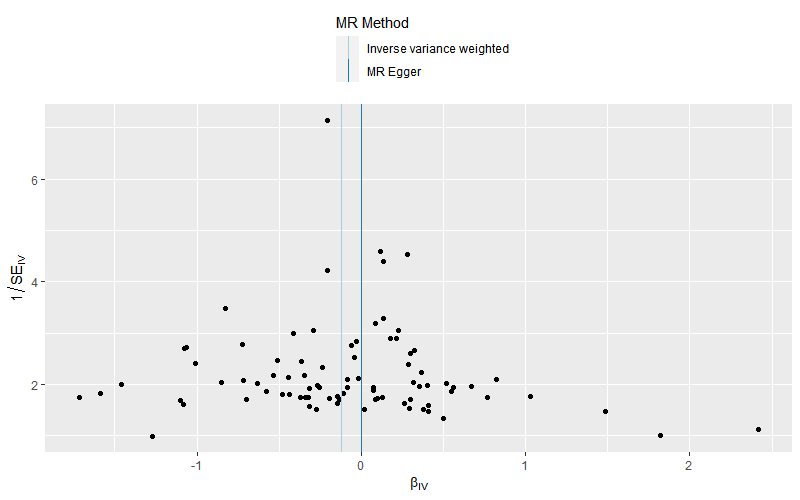

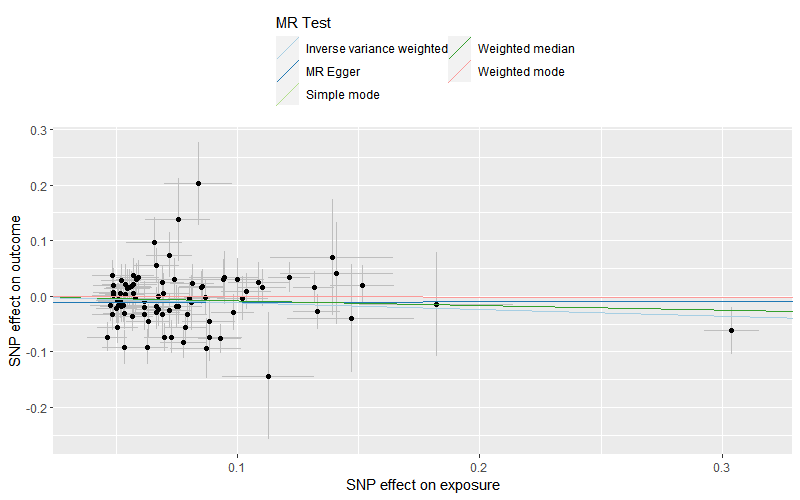


**Supplementary Figure S11.** Forest plot, leave-one-out plot, funnel plot and scatter plot for the MR analysis inferring the causal effect of lung adenocarcinoma on hypothyroidism


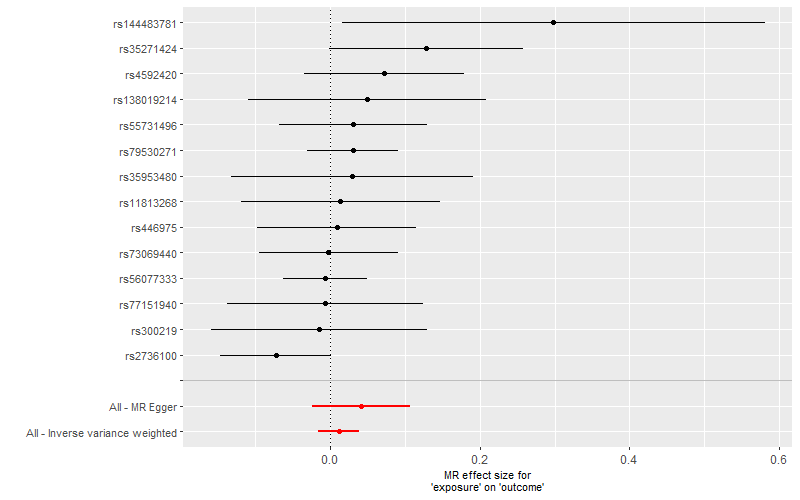

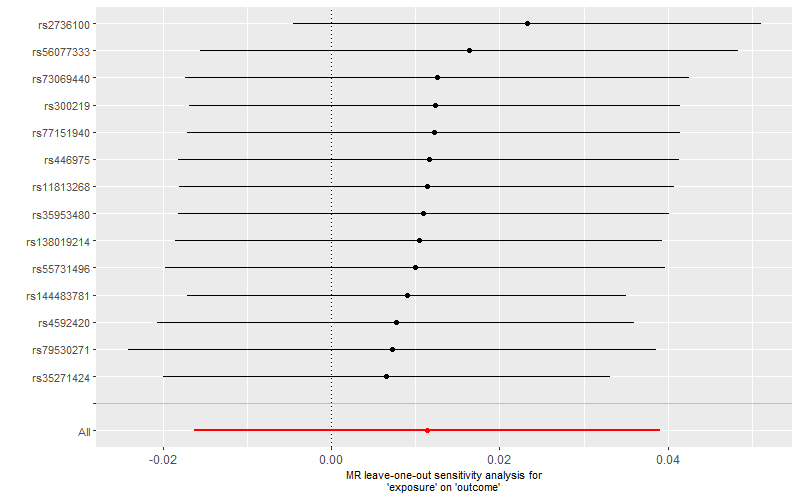

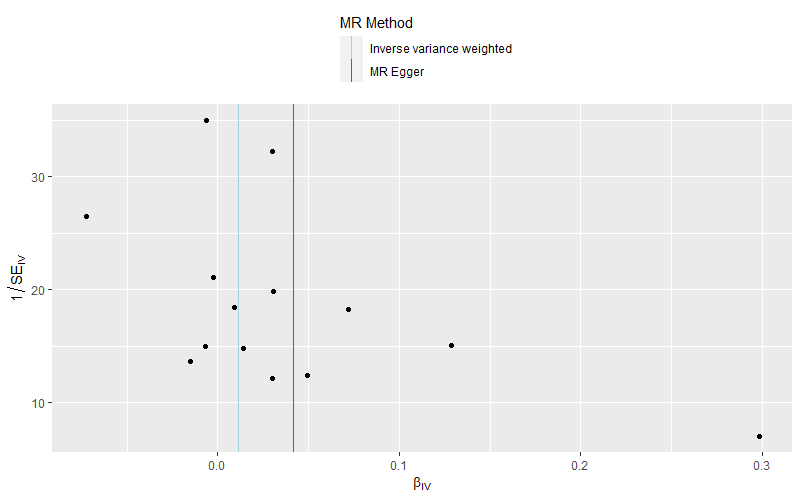

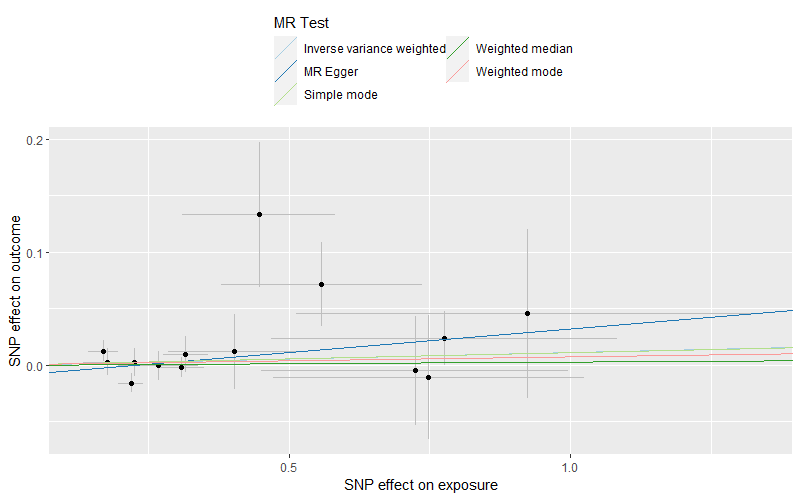


**Supplementary Figure S12.** Forest plot, leave-one-out plot, funnel plot and scatter plot for the MR analysis inferring the causal effect of Squamous cell lung cancer on hypothyroidism


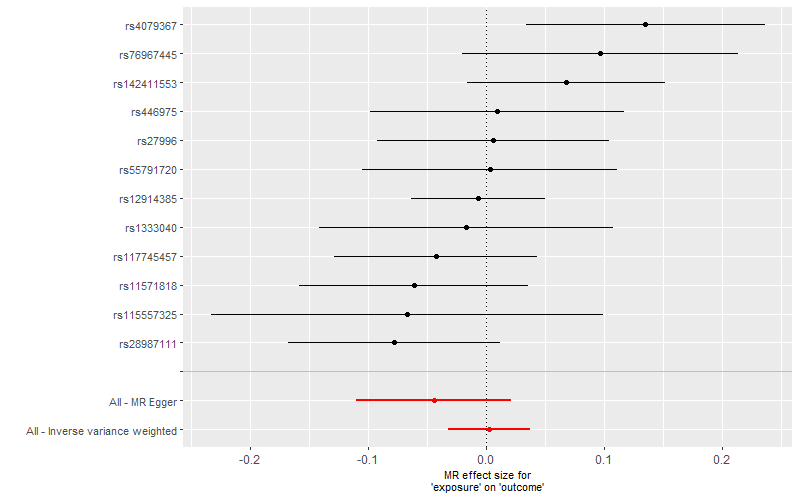

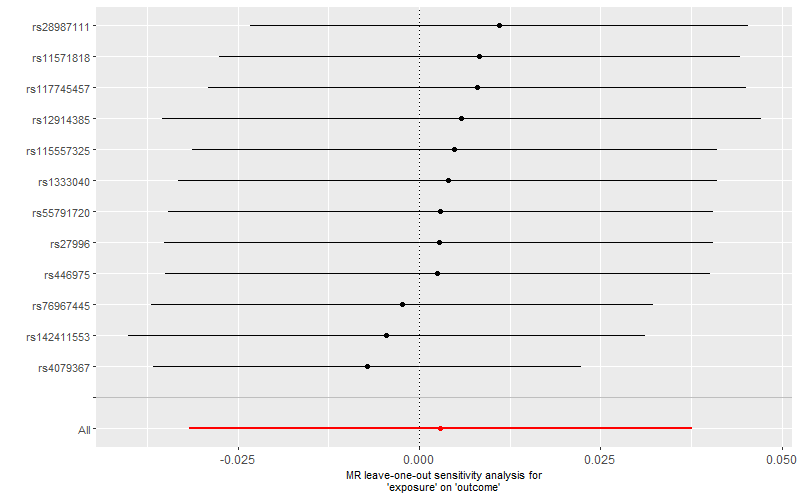

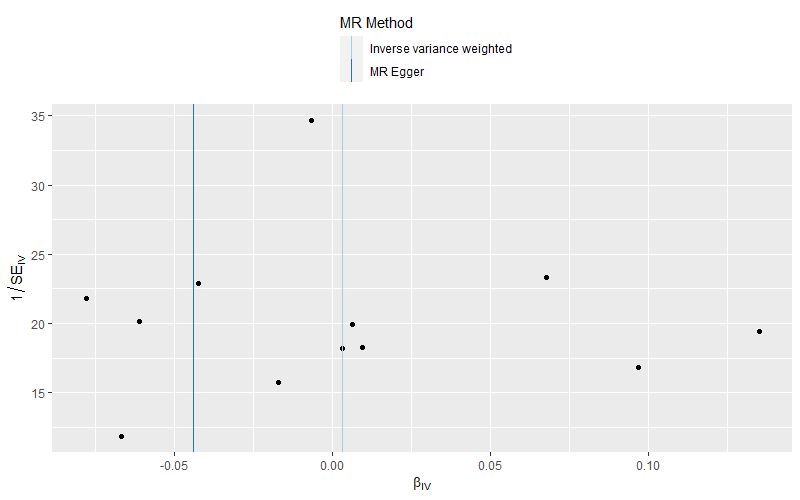

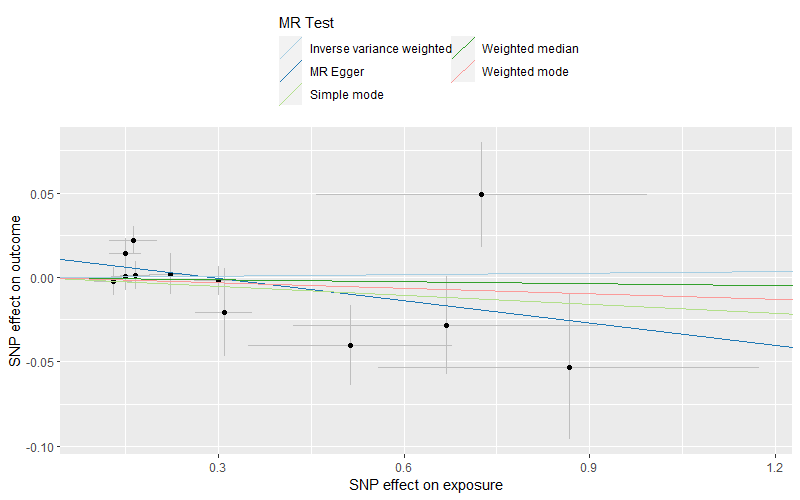

Supplement: Supplementary file 1 — Supplementary Material 1 [file 12890_2023_2588_MOESM1_ESM.docx]
